# How best to use photons

**DOI:** 10.1107/S2059798319003528

**Published:** 2019-03-19

**Authors:** Graeme Winter, Richard J. Gildea, Neil G. Paterson, John Beale, Markus Gerstel, Danny Axford, Melanie Vollmar, Katherine E. McAuley, Robin L. Owen, Ralf Flaig, Alun W. Ashton, David R. Hall

**Affiliations:** a Diamond Light Source, Harwell Science and Innovation Campus, Didcot, Oxfordshire OX11 0DE, UK

**Keywords:** radiation damage, data collection, data processing, data analysis

## Abstract

Strategies for collecting X-ray diffraction data have evolved alongside beamline hardware and detector developments. The traditional approaches for diffraction data collection have emphasised collecting data from noisy integrating detectors (*i.e.* film, image plates and CCD detectors). With fast pixel array detectors on stable beamlines, the limiting factor becomes the sample lifetime, and the question becomes one of how to expend the photons that your sample can diffract, *i.e.* as a smaller number of stronger measurements or a larger number of weaker data. This parameter space is explored *via* experiment and synthetic data treatment and advice is derived on how best to use the equipment on a modern beamline. Suggestions are also made on how to acquire data in a conservative manner if very little is known about the sample lifetime.

## Abstract 2

Different modes of data collection are explored, and the effect of flux and multiplicity on the resulting quality of the data set is discussed. Advice is offered on how to collect data in the absence of prior knowledge of the sample.

## 1. Introduction   

The principal limit on the completeness and accuracy of crystallographic data from third-generation synchrotron sources is often sample lifetime, *i.e.* radiation damage. With CCD detectors this presented a specific challenge: to obtain sufficiently strong data to overcome detector read-out noise whilst obtaining a complete data set, ideally to the highest possible resolution. Strategy programs such as *BEST* (Popov & Bourenkov, 2003 ▸) were developed with exactly this challenge in mind. With the advent of photon-counting detectors, however, the possibility arises of recording far weaker data and instead relying on multiplicity of measurements to obtain improvements to the quality of the data, rather than increasing photon counts for individual observations. Therefore, this raises the question of how best to use the photons that may be scattered within the lifetime of the sample.

While software exists which may estimate the lifetime of samples given a detailed knowledge of the beamline and sample composition (Murray *et al.*, 2004 ▸; Zeldin *et al.*, 2013 ▸), and strategy programs exist to exploit this information, these are sensitive to the initial input and require a detailed knowledge of the beam profile, intensity and sample composition. The aim here is to arrive at a protocol that may be used in the absence of this preparation but should still arrive at a good quality data set, *i.e.* a general strategy rather than a sample-specific one.

In arriving at such a strategy, there are four specific questions that must be answered.

(i) Is a larger number of weak observations equivalent to a smaller number of stronger observations with the same total photon counts?
(ii) If very weak data are recorded, are they useful?
(iii) Given a reasonable multiplicity of observations, how can the presence of radiation damage be detected, and where is the optimum point to ‘truncate’ the data set?
(iv) Given data from multiple samples, is it better to combine weak complete sets or stronger partial ones?

These questions will be considered in sequence, with example data sets to consider each point. Extensive use will be made of merging statistics, and the reader is directed to https://strucbio.biologie.uni-konstanz.de/ccp4wiki/index.php/R-factors for a refresher, if needed.

## 2. Strength versus multiplicity   

Any data-collection strategy that depends on multiplicity of measurements must first ask if, in the absence of significant radiation damage, the results of a high-multiplicity low-dose experiment are equivalent to the same number of photons scattered from the same crystal over fewer reflections. Recording fewer, stronger reflections (whilst still a complete set) may be an effective strategy if (i) the sample lifetime is well known, (ii) data size (disk storage) is a factor and (iii) acquisition time is a major consideration. If the sample lifetime is not well known, for example a novel protein where the sample behaviour has not been previously characterized, there is a strong argument for a conservative approach to data collection, *i.e.* recording more data with a lower intensity beam, such that in the event of radiation damage being found the data may be cut back *post mortem*, reducing multiplicity but ideally not completeness.

To address this question, data were recorded on Diamond Light Source beamline I24 from three cubic insulin samples, deliberately grown to be comparable in size to the beam (details in Appendix *D* in the Supporting Information). The total dose (*i.e.* full-beam seconds) for each was kept as close as possible to constant, as well as keeping it low to reduce effects of damage, resulting in relatively weak but comparable data sets – the data-collection parameters are listed in Table 1 ▸. All data were recorded with an exposure time of 20 ms per frame at 0.9686 Å, with the total rotation and transmission adjusted to give approximately the same total dose of around 0.16 MGy, as estimated by *RADDOSE-3D* (Zeldin *et al.*, 2013 ▸). For each sample, multiple data sets were recorded with varying total rotation and transmission, in a randomly selected order, with the first scan repeated at the end to allow direct comparison. In all cases no signs of significant radiation damage were detected, and the results of structure refinement were comparable.

**Figure 1:**
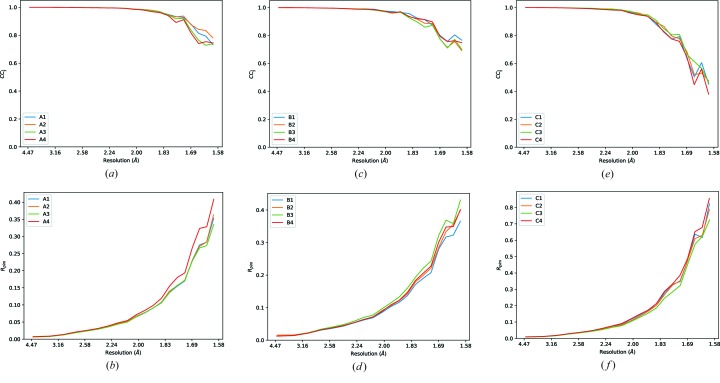
Merging statistics for 12 comparable data sets from three samples (A, left; B, middle; C, right) where the total number of scattered photons was kept approximately constant while the transmission and total rotation range varied to assess the effects on the total data quality.

All had around 0.4 full-beam seconds of data collected, around 1.2 × 10^12^ photons. While the *R*_merge_ values vary as expected, the *R*_p.i.m._ values are relatively consistent (Fig. 1 ▸). An additional sample was collected where the total dose was around eight times higher, with the corresponding improvement in *R*_p.i.m._, indicating that the dominant factor in the precision of the measurements was the total scattered photons. As such, there is no evidence that recording higher multiplicity weaker measurements has any detrimental effect on the overall data quality or final resolution limit. In particular, the final resolution limits as estimated by CC_1/2_ ≃ 0.5 for each of the data sets recorded on the three crystals were comparable. It is important to note that there are practical limits to this, as the data must be strong enough that spot finding and indexing remain successful.

## 3. Transmission ladder   

In many cases the expected lifetime for a sample will not be known *a priori*. However, there will usually be fairly well known extrema, for example a minimum and maximum typical lifetime, which may differ by one or more orders of magnitude. In this situation, a conservative strategy for data collection could be to acquire first an exceedingly weak full rotation, *i.e.* well below an anticipated lifetime dose of the sample, then the same rotation with 4, 16 and perhaps 64 times the dose – in principle doubling the Poisson-derived *I*/σ(*I*) each cycle. It is highly likely that the later runs will have substantial radiation damage, however if this is observed, the previous run should always give complete data, or as complete as possible given the geometric constraints. The earlier low-dose data may also be suitable for molecular replacement or substructure determination, where subsequent (potentially somewhat damaged) data could be more suitable for structure refinement as a higher resolution may have been achieved. Conversely the stronger but radiation-damaged data could be useful for determining an initial sample orientation, which could then be used to process the weaker data.

### 3.1. Difference maps for ligand binding   

Ligand-binding studies for drug discovery is a common use for data collection at synchrotron sources. In such cases the majority of the atomic positions are well known, so even imprecise data may be adequate to observe the differences between the sample under study and the existing model, thus showing any ligands. This may be demonstrated by taking a sequence of data sets from a sample with a ligand, with a range of transmissions, and computing difference maps for each.

**Figure 2:**
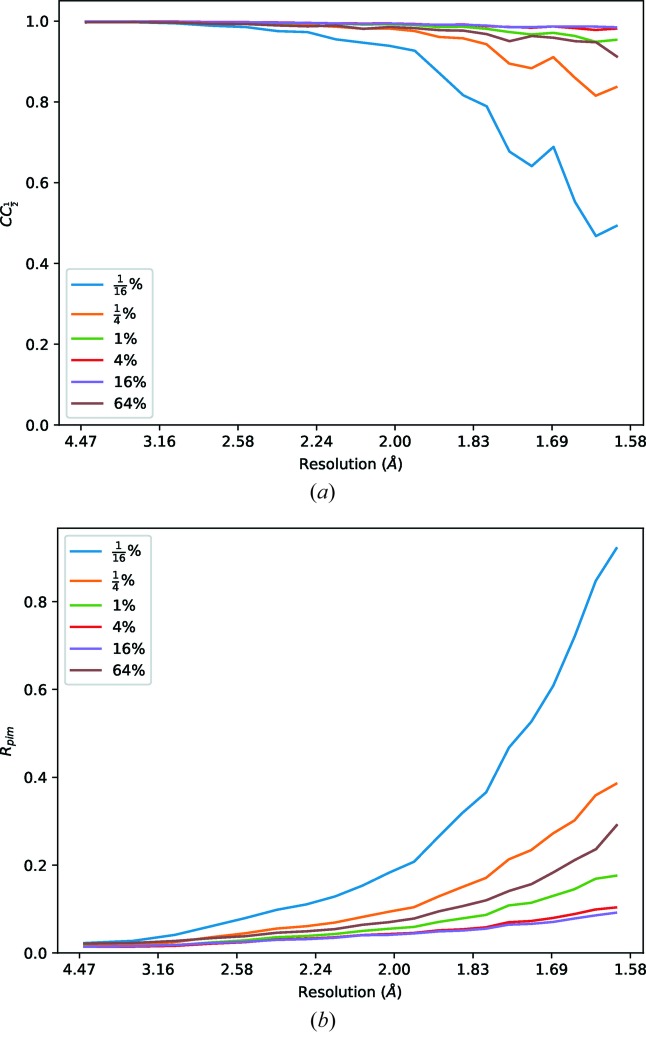
Merging statistics for thaumatin data sets recorded with transmissions from 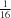 to 64%, processed to a fixed resolution of 1.6 Å. Clearly the weakest of these data are suffering from poor precision in the intensity measurements, which rapidly improve as a greater dose is applied. There is, however, a point of diminishing returns between 1 and 16% where radiation damage becomes a greater factor in data quality than counting statistics.

Data were collected at Diamond Light Source beamline I03 from a thaumatin crystal prepared following standard protocols with tartrate in the crystallization conditions. Each data set was recorded as 3600 × 0.1° images with 40 ms exposure period, with transmissions as close as possible to 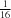, 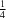, 1, 4, 16, 64% (*i.e.* ∼1 × 10^9^ to ∼1 × 10^12^ photons s^−1^) for a total of six runs. The steps in transmission were chosen to give an approximate doubling of *I*/σ(*I*) due to counting statistics (Fig. 2 ▸ and Table 2 ▸).

**Figure 3:**
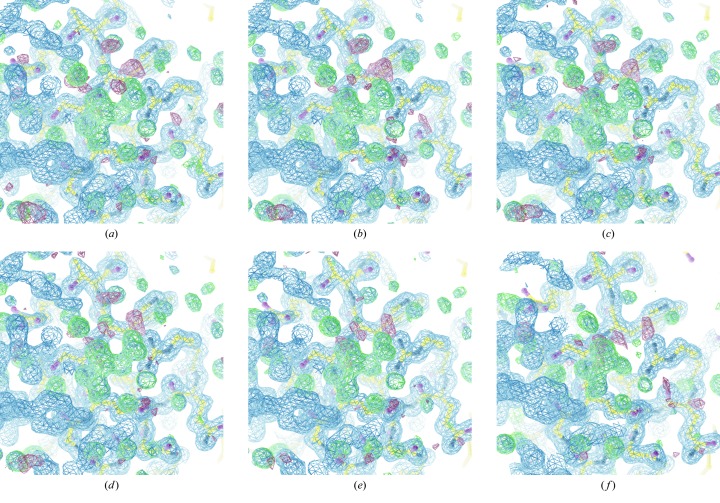
Difference maps (rendered at 3σ) derived from thaumatin data, showing the tartrate molecule from the crystallization conditions, for data recorded with transmission from 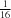 to 64%. Signs of radiation damage are clearly visible in the electron density in the last of these data sets. Of particular interest is the similarity in the maps (*b*)–(*e*): by eye there is very little difference in the maps despite the factor of 64 difference in transmission used.

Each data set was processed independently with *xia2*/*DIALS* (Winter, 2010 ▸; Winter *et al.*, 2018 ▸) to a fixed resolution of 1.6 Å, and *DIMPLE* (http://ccp4.github.io/dimple/) was run to compute a difference map, using a model of thaumatin without tartrate present. As can be seen in Fig. 3 ▸, even though the merging statistics are very poor from the weakest data set, the map shows clear difference density which is reproduced by the subsequent data sets. The structure refinement also shows a good agreement between the model and the data, though the stronger data sets before radiation damage becomes apparent give slightly improved statistics. 

This clearly demonstrates that though the data are very weak and show rather high merging residuals, the averaged data are nevertheless useful for ligand identification, and can be acquired with as little as one tenth of a full-beam-second worth of exposure. While thaumatin crystals are well known to be robust in the beam, clear signs of radiation damage such as a significant fall-off in resolution were visible in the 16% and 64% data sets. The question of radiation damage will be revisited in Section 5.

### 3.2. Symmetry determination and molecular replacement   

Traditional data-collection strategies from *e.g.* *EDNA* (Incardona *et al.*, 2009 ▸) rely on acquiring a small number of ‘screening’ images from which the lattice symmetry is derived *via* indexing. In the majority of cases this will result in the correct lattice, however, in some circumstances accidental symmetry in the unit-cell parameters (*e.g.* an orthorhombic primitive lattice with *a* = *b*) may give misleading results. This may only be discovered subsequently once a full data set has been collected and the integrated intensities have been analysed. Such an analysis may however be successfully performed with a very low dose data set. Similarly, molecular replacement is principally dependent on the low-resolution (from ∞ to ∼4–2.5 Å) data (Evans & McCoy, 2008 ▸), so intensities resulting from a low-dose sweep may be useful for assessing molecular replacement models.

To demonstrate this, data were collected from four crystals of cyclin dependent kinase 2 (CDK2) kindly provided by Arnaud Basle of Newcastle University, UK, and stepped transmission data collected as for thaumatin above. Although the crystals have orthorhombic *P*2_1_2_1_2_1_ symmetry, the unit-cell *b* and *c* axes are very similar in length, giving a pseudo-tetragonal lattice. Analysis of the intensities with *POINTLESS* (Evans, 2011 ▸) – even of the very weakest data set – clearly shows the presence of three twofold axes and the absence of the fourfold (Table 3 ▸). As such, even if the stepped transmission approach is not used for data collection, there may be substantial value in collecting a relatively complete low dose data set rather than a sequence of single images separated in ω for screening. The full processing results from all sets for all crystals are shown in Table S9 in the Supporting Information.

After processing, the data were taken forward to molecular replacement with *PHASER* (McCoy *et al.*, 2007 ▸) using as a search model PDB entry 1hck (Schulze-Gahmen *et al.*, 1996 ▸). Despite the low overall *I*/σ(*I*) of the weakest data (∼5) molecular replacement was successful in every case, as judged by *TFZ* scores in the range 46.7–59.6. As such, even very weak or low-dose data may be useful for assessing the crystal symmetry and testing molecular replacement solutions prior to acquiring full data sets for final structure determination and refinement, though in this case even the weakest data set gave a good refined structure.

### 3.3. Exploration of parameter space with insulin   

Data were collected from four cubic insulin crystals on Diamond Light Source beamline I03. Each data set consisted of 4800 images at 0.15° per 0.04 s, at a wavelength of 1.2 Å, 6.25% transmission (∼3.1 × 10^11^ photons s^−1^) and at a distance such that the inscribed circle on the detector was at 1.4 Å. Despite the low transmission, each data set showed signs of very mild radiation damage (shown in Appendix *D* in the Supporting Information). However, each data set also contained sufficient anomalous signal to allow phasing *via* S-SAD with *SHELXC/D/E* (Sheldrick, 2010 ▸) making them useful for exploring parameter space.

For a given total dose, the choice will be between strength and multiplicity, as discussed earlier in Section 2. Here, however, this may be explored in more depth by taking either subsets of the data or by applying *a postori* transmission adjustment by digital attenuation.

#### 3.3.1. Digital attenuation   

In a monochromatic synchrotron beamline, the photon flux is controlled (for a given source configuration) by attenuator foils or wedges, which absorb a predictable fraction of the primary beam. Obviously the absorbed photons could have contributed to background, Bragg diffraction or simply passed through the sample, so the filter transmission has the overall effect of approximately scaling the image. It is important to note that this is not a simple scaling, since all processes involved are stochastic.

**Figure 4:**
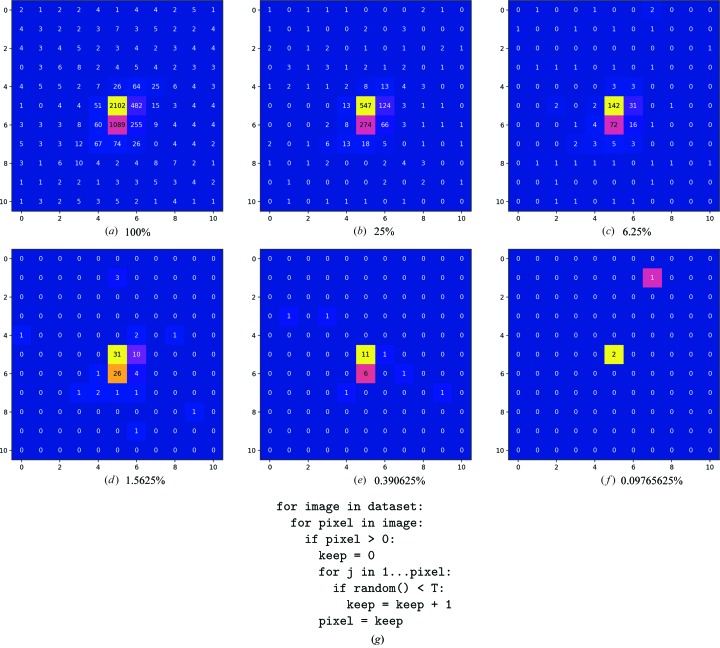
Examples of a digitally attenuated diffraction spot for transmissions 1 to 4^−5^, and a scheme showing the mechanism for digitally attenuating data in place, for a transmission factor *T*. The command line for running *DIALS* implementation included in Appendix *D* in the Supporting Information.

To reproduce this process *in silico*, care must be taken to ensure the stochastic processes are reproduced. The scheme in Fig. 4 ▸, derived from Section 10 of Waterman *et al.* (2016 ▸), is designed to reproduce this: for each count recorded on every pixel of every image a random value is drawn from [0.0, 1.0).^1^ If this random value is less than the desired transmisson factor *T*, the count is kept in the data, otherwise it is rejected. This will therefore maintain the statistical structure of the data, whilst reducing the intensity in the background and reflections equivalently. This is illustrated in Fig. 4 ▸ for one reflection on one image. Clearly, any radiation damage present in the original data will continue to be present in the attenuated data. 

Use of this attenuation scheme will therefore allow a fairer comparison of the effects of transmission with the size of the data set, though radiation damage is not taken into consideration. This scheme is only applicable to data from a photon-counting pixel-array detector, since the events must be individually recorded and uncorrelated with one another.

#### 3.3.2. Results   

**Figure 5:**
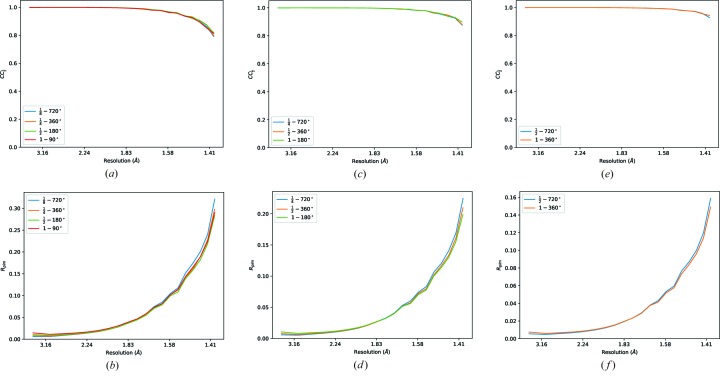
Merging statistics for data derived from the first insulin crystal, with digital transmission applied. The data are indexed by the transmission factor from 1 to 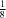 (*i.e.* equivalent photon flux from 3.1 × 10^11^ to 3.9 × 10^10^ photons s^−1^) and the total rotation included (*i.e.* all 720°, first 360°, 180° and 90° of the data). Data sets included in each plot are in principle comparable, as the product of the rotation and transmission factor is constant.

The merging statistics for each combination of transmission and subset of the data are shown for the first insulin crystal in Table 4 ▸ and (Fig. 5 ▸). Data for all crystals are included in the Supporting Information. In the table, each row in principle corresponds to comparable data sets, *i.e.* the same total photon count, though data sets with a wider rotation range will include more of the small amount of radiation damage present in the original data. As may be expected, the overall *R*_meas_ value for each of the transmission values remains approximately constant, however between transmissions the values change by a smaller factor than would be expected from counting statistics alone. The total summation-integrated counts for each processed data set behave as expected, deviating only a couple of percent from the desired total, which should be expected as the illuminated volume of the crystal will vary as the crystal is rotated.

Based on the merging statistics alone, for a given total dose the best overall *R*_p.i.m._ comes from the higher multiplicity weaker data, which is slightly counterintuitive given the radiation damage. The outer shell *R*_p.i.m._ values, however, are generally better for the stronger, lower multiplicity measurements. This may reflect the increased sensitivity of high-resolution data to radiation damage, but could also reflect the increased sensitivity of weak, high-resolution data to systematic effects: a greater number of unique paths through the crystal will increase the spread of absorption paths sampled and therefore the spread relating to insufficient fidelity in absorption modelling, as the large samples (around 100 µm) and the wavelength of 1.2 Å are sufficient to give around a 5% chance of photon re-absorption based on a linear attenuation coefficient from *RADDOSE-3D* of 5.83 × 10^−4^ µm^−1^. While this may negatively affect the *precision* of the high-resolution intensities, it is not clear that this would affect the *accuracy* of the averaged intensities. The merging statistics may therefore be inconclusive in deciding on a high-multiplicity or high-dose strategy. Similar conclusions can be drawn for all four crystals, from results shown in the Supporting Information.

#### 3.3.3. Substructure determination   

For most users the most useful measure of data quality is whether the data answer the experimental question. For ligand-binding studies this is a relatively low bar, as much of the structural information is known *a priori*. For experimental phasing, however, almost all of the structural information is derived from the experimental data. For phasing with *SHELXC/D/E*, the *SHELXE* phasing step is particularly effective if the data are high resolution and the solvent fraction is large: both of which apply to these insulin data where the solvent fraction is around 64%. Therefore the success of the substructure determination will be used as the metric for data comparison here.

**Figure 6:**
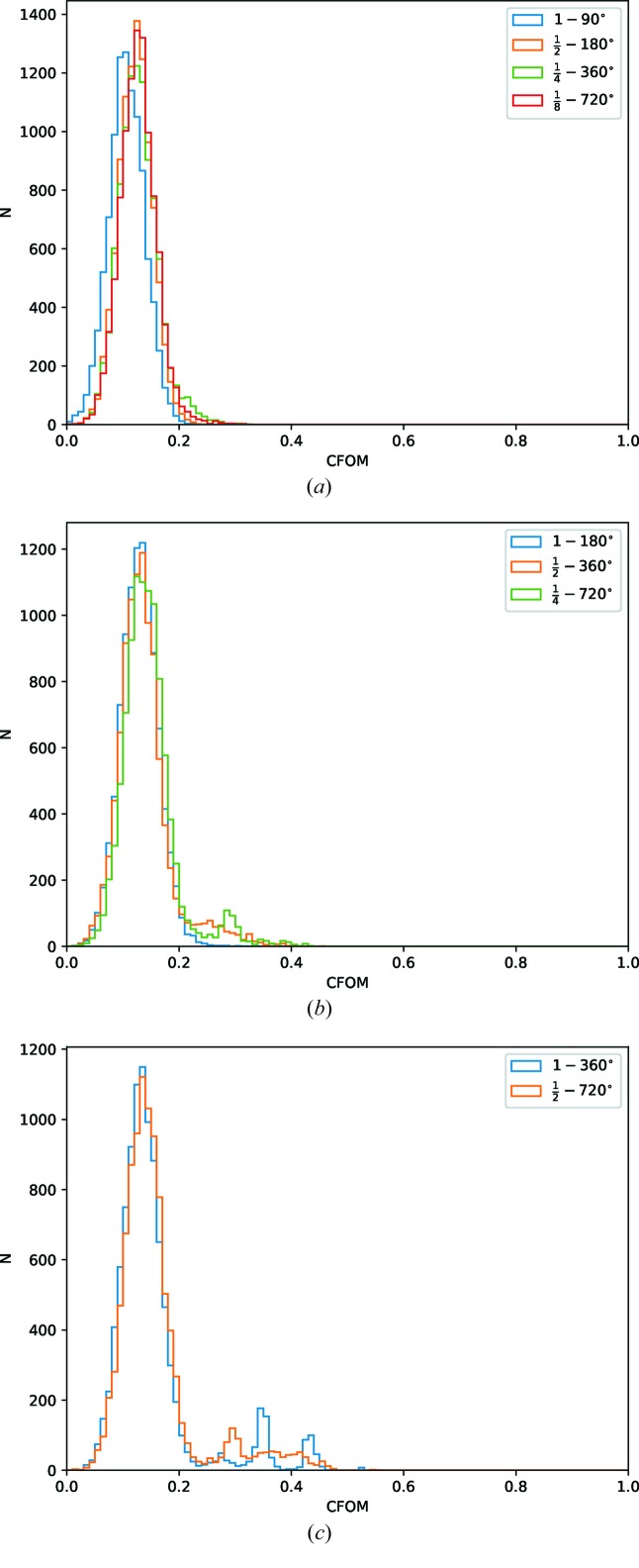
Histograms of combined figure of merit (CFOM = CC_all_ + CC_weak_) from *SHELXD* for 10 000 trials for comparison data sets with 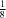 original total photon count (*a*) 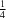 (*b*) and 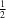 (*c*).

For the substructure determination a fairly standard *SHELXC/D* script was run, with 10 000 trials^2^ using data to 1.9 Å, seeking three disulfides, and histograms of the combined figure of merit (CFOM = CC_all_ + CC_weak_) used to assess success. From Fig. 6(*a* ▸), it is clear that substructure determination was generally unsuccessful for the data sets with 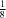 of the original photon count. Manual verification of the subsequent phasing with *SHELXE* confirmed that the overall phasing process was unsuccessful. For the data with 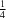 of the original photon counts [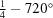, 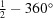 and 1 − 180°; Fig. 6(*b* ▸)] some of the trials gave potentially useful solutions for the 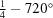 and 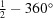 sets. Subsequent phasing with *SHELXE* showed a substantial contrast difference between the hands and interpretable maps from both sets, with only 1000 trials run. For the last comparison set with half of the original photon count [Fig. 6(*c* ▸)] both sets unsurprisingly gave good solutions. Inspection of the histograms suggests roughly the same number of useful solutions, indicating that the two sets are effectively equivalent in terms of substructure determination.

### 3.4. Resolution limits for weak data   

**Figure 7:**
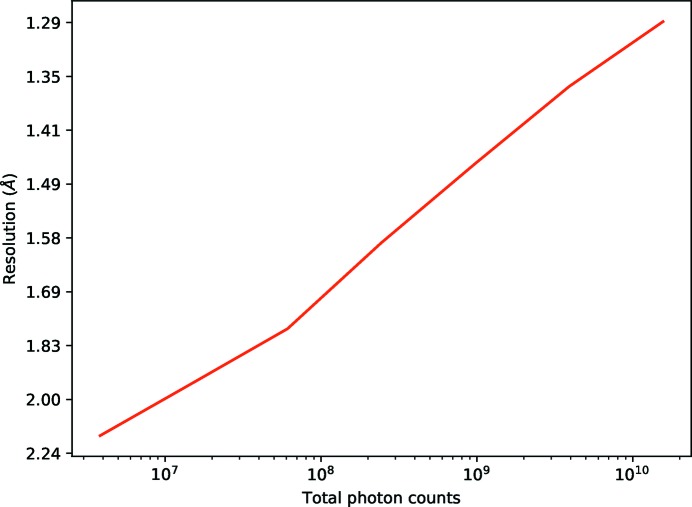
Resolution (derived from CC_1/2_ ≃ 0.5) versus total counts for digitally attenuated cubic insulin data, for attenuations in the range 0.0244% to 100%. The corresponding resolution limits increase from 2.15 to 1.29 Å.

A clear advantage of using a higher total dose is that the data are generally significant [as measured by CC_1/2_ or *I*/σ(*I*)] to a higher resolution as the effects of random errors are reduced. Digital attenuation can be used to show that even very weak data can be sensibly interpreted and arrive at the correct symmetry albeit with substantially poorer merging statistics. 360° of data were taken from cubic insulin crystal 3 and attenuated by factors of 4^−*n*^ for values of *n* in the range 0–6 (*i.e.* from 100% of the photons to 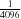
%). Fig. 7 ▸ shows the total counts in the data set and the processed resolution using *xia2*/*DIALS* (full statistics shown in the Supporting Information). The trends as presented are remarkably linear, as the resolution limits are well within the linear regime of the Wilson plot, so doubling the *I*/σ(*I*) of the data will give a corresponding increase in the 1/*d* ^2^_min_. The gradient of this line depends on the overall *B* factor of the crystal. The corollary of this is that an increase in transmission of around 256 was necessary to improve the resolution limit by 0.5 Å. Clearly this behaviour is sample dependent, and most samples diffract rather less well than insulin, with a higher intrinsic *B* factor. This however emphasises the value of using lower transmissions: the reduction in resolution for using a quarter of the dose will, in general, be much more modest, whilst the damage will be massively reduced. Recording data from mutiple isomorphous samples may be a practical way of improving the resolution, as the total number of scattered photons can increase without increasing the damage to individual samples. Similar results to those presented here have been reported in Yamamoto *et al.* (2017 ▸), though there the emphasis was on achieving resolution *via* high-flux beamlines whereas here we highlight the massive increase in photon count necessary to achieve a modest increase in resolution.

## 4. Diminishing returns   

In the absence of radiation damage, increasing the multiplicity of observations will always improve the precision of the average intensity measurements, all other things being equal. Indeed, collecting high multiplicity data from one or several crystals is a well established mechanism for improving data quality (see *e.g.* Liu *et al.*, 2011 ▸). If, however, the repeated measurements are through the same path through the crystal and on the same detector position, they may suffer the same systematic errors and therefore do little to improve the accuracy of the average measurements. Also, in reality, radiation damage is rarely undetectable for very high multiplicity data sets, as shown from the following.

**Figure 8:**
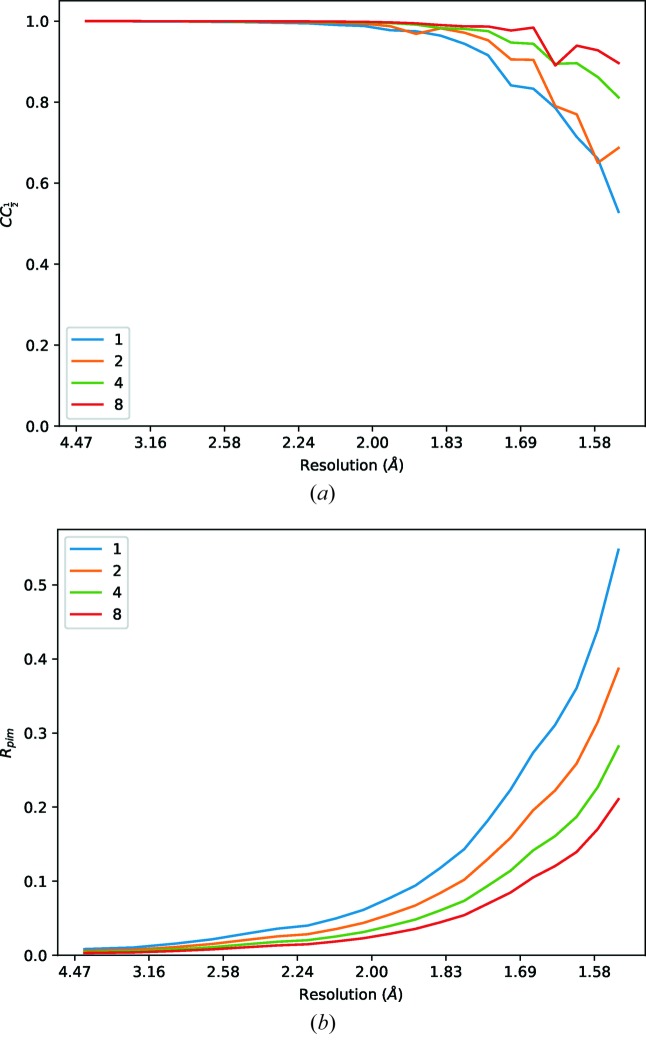
Merging statistics for weak thermolysin data sets, for one, two, four and eight double rotations (*i.e.* 720° data sets) at very low transmission.

**Figure 9:**
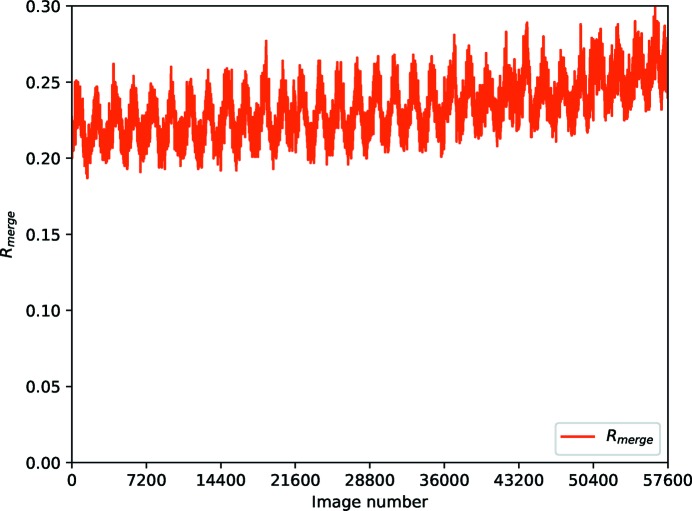
*R*_merge_ versus frame number for 8 × 720° data sets, showing a steady increase in the statistic alongside a periodic variation due to illuminated volume.

Data were collected from a standard thermolysin test crystal with very low transmission (0.05% giving ∼2.5 × 10^9^ photons s^−1^) on Diamond Light Source beamline I03. Eight data sets each consisting of 7200 × 0.1° images were recorded, and the structure refined against the first set (Winter *et al.*, 2018 ▸) and re-refined against data consisting of the first one, two, four and all eight data sets (Table 5 ▸). Although the *R*_merge_ is very high, corresponding to the very weak individual observations, the multiplicity is extremely high (from 70 to around 600-fold). As may be seen from Fig. 8 ▸, the *R*_p.i.m._ and CC_1/2_ values improve for each data set, roughly in line with the multiplicity of measurements. There are however signs of modest radiation damage (Fig. 9 ▸). The results of refinement do not show such substantial improvements, suggesting that the precision of the measurements (*i.e.* number of scattered photons) is not a significant factor (in this case) in the overall quality of the final model, comparable with the outcomes in Section 3.1.

## 5. Radiation damage   

With modern third- and fourth-generation synchrotron sources, radiation damage is the greatest limit on collecting data. Most obviously the problem of damage will become apparent as poorer diffraction on later images in the data set. By this time there is clearly nothing that can be done to correct the experiment, however it may be possible to recover something from the data if a high multiplicity strategy has been employed. Alternatively, this outcome may be used to give some insight into sample lifetime for subsequent data collections – the so-called ‘sacrificial crystal’ (Leal *et al.*, 2011 ▸). In either case the data should be appropriately analysed to estimate the useful sample lifetime.

### 5.1. Analysis statistics   

**Figure 10:**
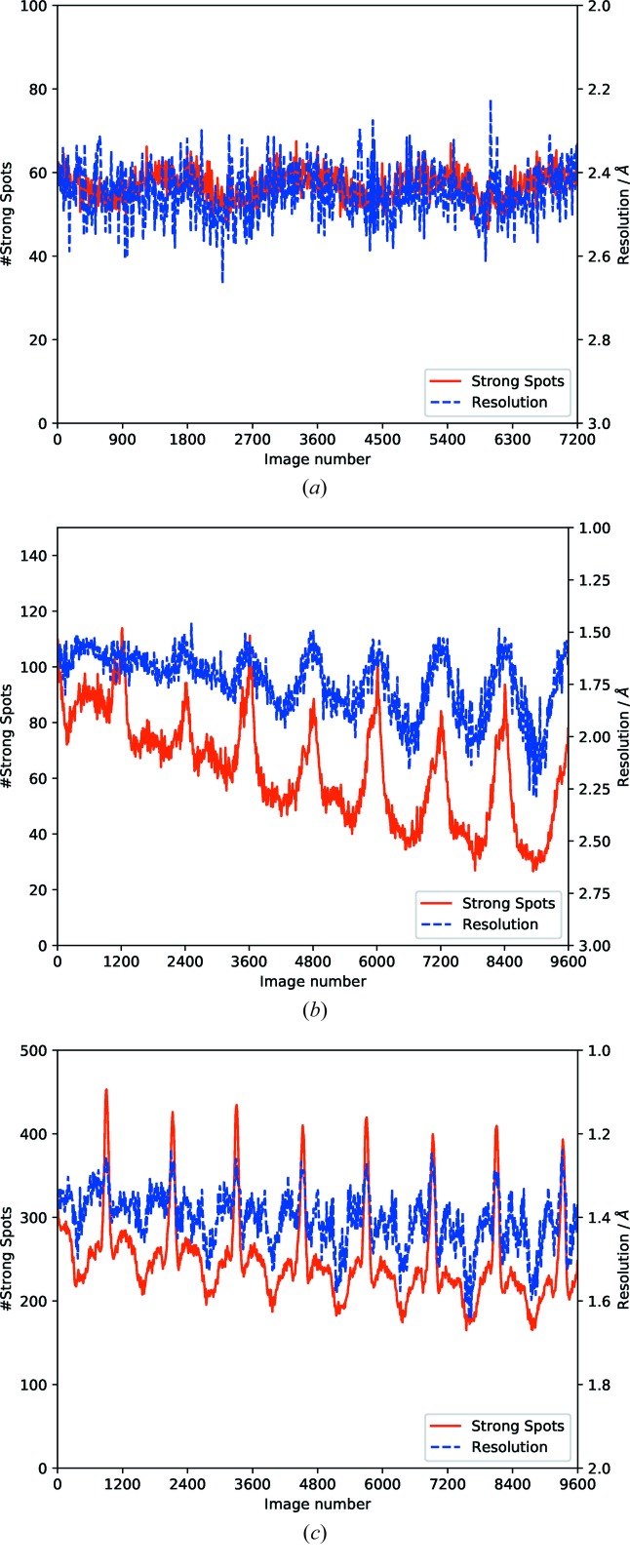
Number of strong spots (red), and estimated resolution (blue), found per image for a number of different samples with varying degrees of radiation damage. (*a*) The first sweep of the weak thermolysin data; though there are some details resulting from the unit-cell dimensions and changes in illuminated volume, the overall trend is level. (*b*) A sample of BRD4, deliberately radiation damaged to indicate the fall off in resolution (blue) and number of strong spots (red). The sinusoidal pattern results from variations in illuminated volume. (*c*) A sample of CDK2, showing a less severe decrease in the strength of diffraction. Once again the ‘shape’ of the curve of strong spots depends on the sample morphology and unit cell.

The most obvious effect of radiation damage during the diffraction experiment is the fall-off in resolution during the data set. This may be determined either by eye, by inspecting the diffraction images, or by using the spot-finding tools in data-processing software. At most facilities some kind of on-line analysis performing spot finding with *e.g.* *DIALS* (Winter *et al.*, 2018 ▸), *DISTL* (Zhang *et al.*, 2006 ▸) or *Cheetah* (Barty *et al.*, 2014 ▸) will give feedback on the number of strong spots and an estimate of the resolution, sampled at points throughout the data set. While the interpretation of this feedback may be complicated by the effects of diffraction anisotropy, poor sample centering, differing unit-cell lengths and ‘fresh’ crystal being rotated into the beam, the idea that the sample at the end of the experiment is isomorphous with the one at the start can be tested. Fig. 10(*a* ▸) shows a case where no radiation damage is apparent, with the first run of thermolysin data from Section 4, with the plot derived from spots found on all images and averaged over ten-image intervals (*i.e.* 1°). While a certain amount of point-to-point variation is obvious, the overall trend is flat as expected, with a modest periodic variation. It is important to note that the resolution value here is a substantial underestimate compared with the final high multiplicity scaled and merged data set.

In cases where the radiation damage is more obvious the fall-off in diffracting resolution can be dramatic. Fig. 10(*b* ▸) shows data collected from a crystal of bromodomain-containing protein 4 (BRD4; Filippakopoulos *et al.*, 2012 ▸) also provided by Arnaud Basle for radiation-damage studies on Diamond Light Source beamline I03. Data were collected with 9600 40 ms exposures at 0.9762 Å with 50% beam (∼3.8 × 10^11^ photons s^−1^) each corresponding to 0.15° of rotation (*i.e.* a total of four full rotations). While there are clearly some interesting features in the diffraction as the sample is rotated, the overall trend is clearly downward after the first eighth of the data set. In this case attempting to recover a complete set from the beginning of the data or collecting from a fresh sample with much lower transmission may be advisable.

In some cases radiation damage may be present but less severe. The third example (Fig. 10*c* ▸) was collected as part of the same lifetime study, from a crystal of CDK2. Data were collected with the same parameters used for BRD4, with a much more modest fall-off in diffraction during the scan, suggesting that a substantial part or indeed the whole data set could be used downstream.

**Figure 11:**
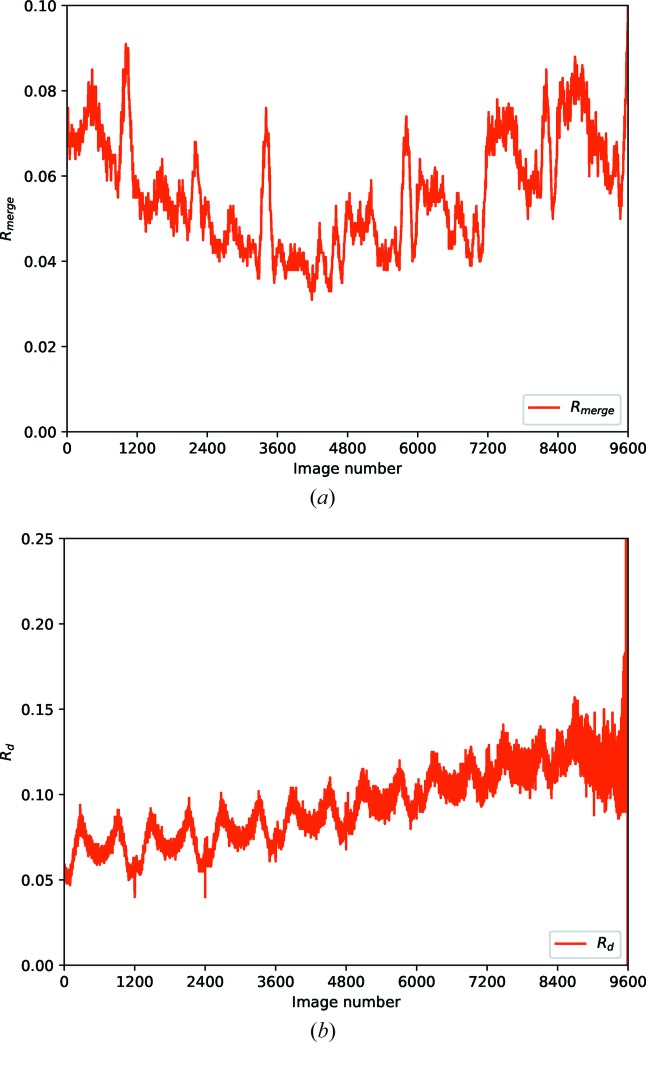
*R*_merge_ versus batch (top) and *R*_*d*_ (bottom) for the CDK2 sample, showing clear signs of radiation damage though no suggestion of the point in the data set where this becomes significant.

After integration and scaling however, the *R*_merge_ versus batch plot from *AIMLESS* (Fig. 11*a* ▸) shows clear indications of radiation damage, with data at the middle of the exposure agreeing better than the extrema (Evans & Murshudov, 2013 ▸). The *R*_*d*_ plot (Fig. 11*b* ▸) (Diederichs, 2006 ▸) shows a clear positive gradient, indicating the presence of radiation damage, though without suggesting a point where this damage becomes problematic. In response to this challenge a new statistic was developed, *R*_cp_, which accumulates the pairwise differences throughout the data set.

### 5.2. *R*_cp_   

The statistic *R*_cp_ was derived from some of the principles behind *R*_*d*_ some time ago (Winter, 2009 ▸) but never formally published though referenced (Evans, 2011 ▸). The derivation started from the principle, analogous to *R*_*d*_, that comparing measurements in a pairwise manner stabilized the statistic with respect to multiplicity of measurements – avoiding the difference between *R*_merge_ and *R*_meas_. However, where
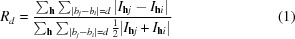
accumulates the differences between measured intensities 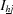 on a baseline of dose (or image number) difference, *R*_cp_ accumulates all differences *up to* this dose or image number, as
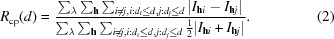
At the time when the statistic was developed (late 2000s) interleaved MAD experiments were *en vogue* for structural genomics, so the intention was to accumulate the statistic across multiple wavelengths following how they were collected. For the most straightforward mode of data collection, *i.e.* high multiplicity experiments as discussed in this section, the interpretation of the statistic is relatively simple: once you have a complete set of observations, the statistic will remain *constant* if the new measurements you are bringing into the data set agree with the existing ones, and will *increase* if they agree, on average, less well than the pairwise observations to date agree. As with all statistics of this nature, it is effectively impossible to disentangle radiation damage from changes in illuminated volume and diffraction anisotropy unless greater than 360° of data have been measured, If you have a sufficient multiplicity of measurements however the trends should be clear.

**Figure 12:**
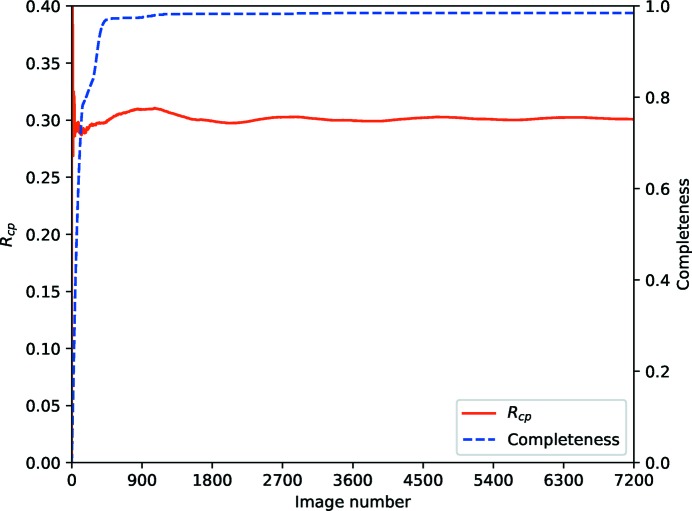
*R*_cp_ and completeness versus batch for the first sweep of weak thermolysin data from Section 4, showing that essentially complete data are present after about 1800 images, and no increase in *R*_cp_ throughout the data set.

Fig. 12 ▸ shows the statistic computed for the thermolysin data used previously. From the completeness curve it is clear that an almost complete data set has been acquired after around 400 images, however a little more anomalous data are acquired after 180° of rotation. Beyond this point, no new measurements are being made, however the repeated observations are in agreement with those measured to this point. At the very earliest stages the statistic is very poorly sampled, so should not be considered reliable (this is comparable with *R*_*d*_ at the far right end of the plot). Including additional measurements will, in this case, improve the precision of the average intensities as expressed in *R*_p.i.m._ as the new observations are drawn from the same population.

**Figure 13:**
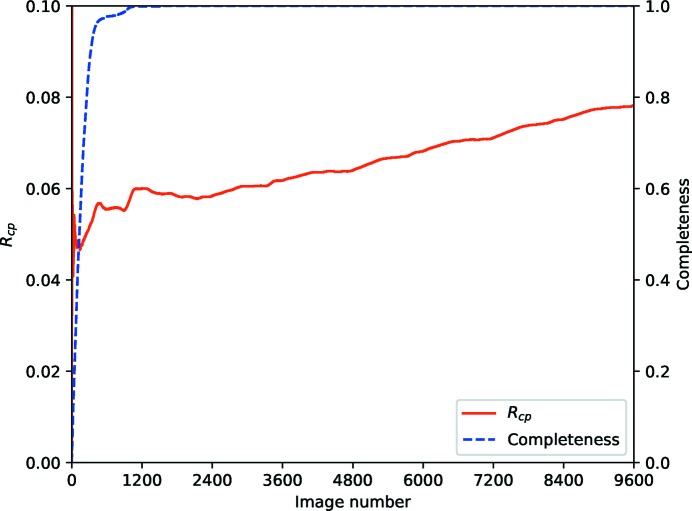
*R*_cp_ and completeness versus batch for CDK2, showing complete data after around 1200 images but substantial increases in the *R*_cp_ statistic after 2400 images (360°).

In the case of the CDK2 data (Fig. 13 ▸) complete data are acquired after around 1200 images (180°) and the *R*_cp_ statistic stays approximately level until about 360° have been collected, after which it increases in a monotonic manner. While including the new measurements *may* improve the *R*_p.i.m._ this will be misleading, as the new measurements are from measurably if slightly different populations. Indeed, as may be seen in Table 6 ▸, including all the measurements in the data set does not give the improvement which could be expected in *R*_p.i.m._, which drops in the outer shell from 0.103 to 0.084 when the quantity of observations is quadrupled. In this case the choice should be made by the experimenter as to how much data to include in the downstream analysis, which may in turn depend on the experimental objectives. For reference, the total dose to the sample from 2400 images (360°) was estimated to be 3.5 MGy, though this is complicated by the sample being substantially larger than the beam.

## 6. Multiple crystals   

The conventional approach to data collection from multiple crystals focuses on constructing a complete set from samples that are highly radiation sensitive. However, as is well established in the literature (see *e.g.* Liu *et al.*, 2011 ▸) combining multiple complete data sets can aid in phasing experiments. By the same token, collecting data from multiple samples also allows the choice on which data to take forward to be made on the basis of downstream analysis. Finally. the intention is to determine structural insight into a biological molecule or complex, rather than a specific sample, so averaging across multiple samples should improve the accuracy of the averaged intensities as sample-to-sample variations in *e.g.* crystal shape and orientation are averaged out.

### 6.1. Sample selection   

**Figure 14:**
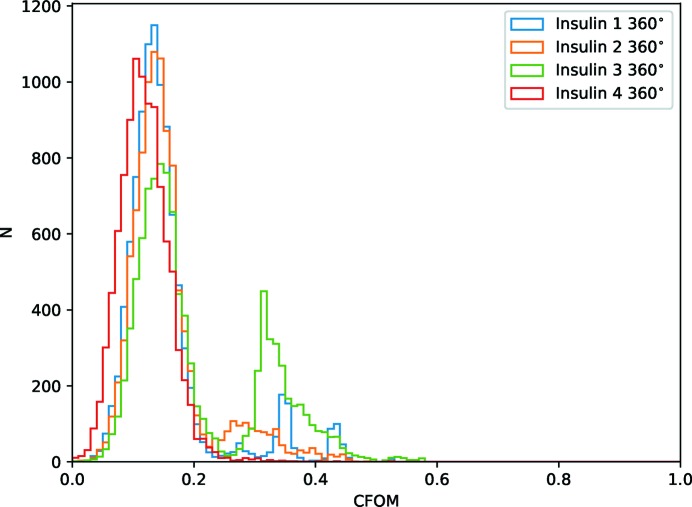
Histograms of combined figure of merit (CFOM = CC_all_ + CC_weak_) from *SHELXD* for 10 000 trials for the first 360° from each of the four insulin crystals. Despite similar merging statistics, the trials for crystal 3 were much more successful than crystal 4.

Before the arrival of photon-counting pixel-array detectors, screening a few samples before selecting the best for data collection was common practice, as acquiring a full data set could take many minutes. With pixel-array detectors on third-generation sources it becomes possible to carefully record a complete 180° or 360° in under a minute, raising the prospect of recording a complete data set from every sample and deciding later how best to use the measurements. The simplest option is to select the data set with the greatest precision to a given resolution limit (*i.e.* lowest overall *R*_p.i.m._) or the strong­est high-resolution data. Table 7 ▸ shows the merging statistics for the first 360° of each of the original cubic insulin data sets used in Section 3.3. While they are similar overall, it may be tempting to select the first as it has the highest overall *I*/σ(*I*), or the second or fourth as they have the highest *I*/σ(*I*) in the outer shell. Substructure determination with the fourth (Fig. 14 ▸) was in fact unsuccessful with 1000 trials, with the third sample having the greatest overall number of successful trials: taking the data forward in parallel was therefore helpful in making a sensible choice.

### 6.2. Combining crystals   

**Figure 15:**
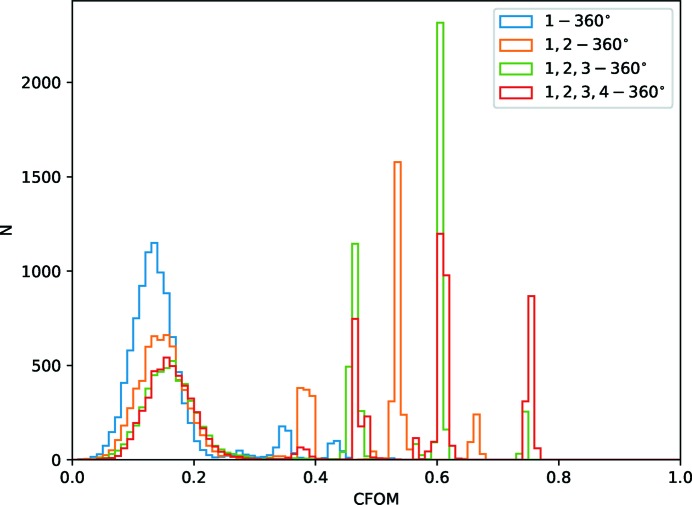
Histograms of combined figure of merit (CFOM = CC_all_ + CC_weak_) from *SHELXD* for 10 000 trials for the first 360° from crystal 1, 1 + 2, 1 + 2 + 3 and 1 + 2 + 3 + 4. As may be expected from the merging statistics, the data from two, three and four crystals give increasingly successful substructure determination.

One well established technique for improving the quality of data sets (Liu *et al.*, 2011 ▸) is to combine the data from multiple samples. An obvious question to ask is whether, in the absence of radiation damage, collecting a given amount of data from multiple samples is equivalent to collecting the same total dose from a single sample. In the general case of course radiation damage will be more substantial with the higher dose, however data can be collected carefully to minimize damage and give data with which this hypothesis can be tested, Table 8 ▸ shows the merging statistics of seven ‘equivalent’ data sets: 360° from each of the four insulin crystals, 180° from 1 + 2 and 3 + 4 and 90° from 1 + 2 + 3 + 4. In all cases the *R*_p.i.m._ and *R*_meas_ are comparable, suggesting that the combined data sets are equivalent *i.e.* that the samples are truly isomorphous. Clearly if radiation damage is not substantial, and the samples are isomorphous, then combining the complete 360° from each set is sensible as this will improve the overall data set. Table 9 ▸ shows the merging statistics for sample 1, then 1 + 2, 1 + 2 + 3 and 1 + 2 + 3 + 4 combined, with the expected improvement in *I*/σ(*I*) and *R*_p.i.m._. Critically, the success rate of substructure trials for phasing (Fig. 15 ▸) improves with the addition of data from each sample, indicating that the combined data set is more useful than any of the individuals as may be expected.

### 6.3. *In situ* data collection at room temperature   

The examples presented so far in this section combined data sets from multiple crystals in order to improve the overall data quality. In some cases, it is simply not possible to collect a complete data set from any one individual crystal, in particular for small, weakly diffracting crystals, or for room-temperature *in situ* experiments (Axford *et al.*, 2012 ▸). In such cases, it is necessary to combine many severely incomplete data sets from many crystals in order to obtain a complete data set. Each individual data set covers a limited region of reciprocal space as a result of small crystal size, radiation damage or limitations of experimental setup (*e.g.* *in situ* data collection).

Processing such data sets presents a number of additional challenges, including symmetry determination (Gildea & Winter, 2018 ▸), scaling, analysis of radiation damage and non-isomorphism (Assmann *et al.*, 2016 ▸), and selection of an optimal data set for downstream phasing and refinement. In this section we describe some of the challenges involved using the example of *in situ* experimental phasing of a proteinase K heavy-atom derivative.

#### 6.3.1. *In situ* experimental phasing of a proteinase K heavy-atom derivative   

*In situ* data collection was performed on both native and heavy-atom derivatives of proteinase K microcrystals. Data were collected on beamline I24 at Diamond Light Source, using a Dectris PILATUS3 6M detector, using a 9 × 6 µm beam with a flux of approximately 2 × 10^12^ photons s^−1^. Data were collected with an oscillation range of 0.1° and exposure time of 0.01 s per image. Data collection was performed across two beamline visits, with 63 and 82 Au-derivative data sets collected across the two visits, giving a total of 145 Au data sets. 50 images (5°) of data were collected per crystal for the first visit of Au data, and 25 images (2.5°) per crystal for the second based on experience from the first visit. In addition, 83 native data sets were collected in a single visit, with 25 images from each.

#### 6.3.2. Data processing   

136 individual Au data sets were successfully processed with *xia2*/*DIALS*, with initial indexing, refinement and integration performed in the primitive triclinic (*P*1) setting. Clustering on unit-cell parameters (Zeldin *et al.*, 2015 ▸) identified a cluster containing 133 data sets in *P*4/*mmm* symmetry, with median unit-cell parameters *a* = *b* = 68.47, *c* = 103.88 Å, α = β = γ = 90°. Analysis with *dials.cosym* and *dials.symmetry*, implementing the algorithms of Gildea & Winter (2018 ▸) and *POINTLESS* (Evans, 2006 ▸) respectively, identified the Laue group as 422. Joint refinement of unit-cell parameters using *dials.two_theta_refine* gave overall unit-cell parameters of *a* = *b* = 68.48, *c* = 103.95 Å, α = β = γ = 90°. Scaling with *dials.scale* gave the merging statistics in Table 10 ▸. Additionally the Au data sets from the two visits were processed independently.

**Figure 16:**
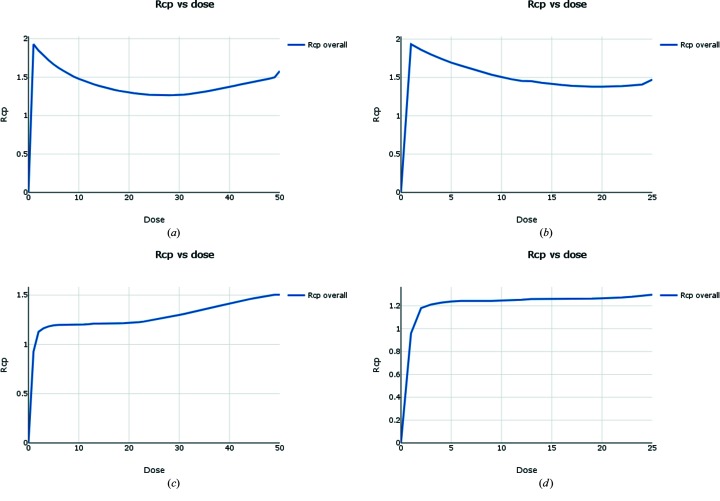
*R*_cp_ versus dose (image number) for Au derivatives of proteinase K, under the assumption that the dose per image is constant across all crystals. (*a*) Data from the first beamline visit display signs of radiation damage after around 25–30 images. (*b*) Data from the second beamline visit display no obvious signs of radiation damage in the plot of *R*_cp_ versus dose (image number). (*c*) Combined data from both beamline visits. A plot of *R*_cp_ versus dose (image number) indicates possible radiation damage after around 25 images. (*d*) Combined data from both beamline visits, using only the first 25 images from each data set. The plot of *R*_cp_ versus dose (image number) displays no obvious sign of radiation damage.

Radiation-damage analysis was performed by calculating the *R*_cp_ statistic presented in Section 5.2, under the assumption that each crystal received an equivalent dose per image (Fig. 16 ▸). From Fig. 16(*a* ▸) it can be seen that after reaching a minimum somewhere between 25 and 30 images, *R*_cp_ begins to climb steadily, suggesting that cutting the data after 25 images may reduce the affects of radiation damage. Therefore, scaling of all 136 data sets was repeated as above, however this time using only the first 25 images of each data set.

Similarly, 76 native data sets were successfully processed, of which 75 remained after clustering on unit-cell parameters, with unit-cell parameters *a* = *b* = 68.43, *c* = 103.87 Å, α = β = γ = 90° after joint refinement with *dials.two_theta_refine*. Merging statistics for all data sets are presented in Table 10 ▸.

#### 6.3.3. Phasing   

**Figure 17:**
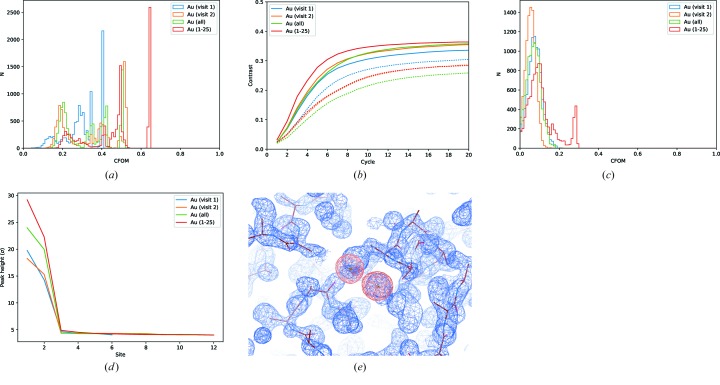
Experimental phasing results for Au derivatives of proteinase K. (*a*) Histograms of combined figure of merit (CFOM = CC_all_ + CC_weak_) from SIRAS substructure determination with *SHELXD* for 10 000 trials, with data from two separate visits individually and combined. (*b*) Map contrast versus cycle number for density modification with *SHELXE*. Solid lines indicate the best hand, while dashed lines correspond to the inverted hand. (*c*) Histograms of combined figure of merit (CFOM = CC_all_ + CC_weak_) from SAD substructure determination with *SHELXD* for 10 000 trials, with data from two separate visits individually and combined. (*d*) Anomalous peak heights calculated with *ANODE*. (*e*) The density-modified (blue) and heavy-atom substructure (orange) phases, contoured at 3σ, and poly-Ala traced model output by *SHELXE* after substructure solution with SIRAS.

Substructure determination using single isomorphous replacement with anomalous scattering (SIRAS) was possible with *SHELXD* (Fig. 17*a* ▸). The heavy-atom derivative data sets were collected across two separate beamline visits. To test the effects of multiplicity on phasing success, substructure determination was attempted separately on data sets coming from a single visit, and on data from both visits combined. Fig. 17(*b* ▸) shows the map contrast versus cycle number after density modification with *SHELXE*. Given the potential for radiation damage in some of the data sets identified above, phasing was also attempted using only data from the first 25 images of each data set. Using only the first 25 images gave improved phases for both heavy-atom substructure and density modification, as judged by the *SHELXD* combined figure of merit (CFOM = CC_all_ + CC_weak_) and *SHELXE* map contrast respectively. The resulting density-modified phases and heavy-atom phases are shown along with the *SHELXE* poly-Ala trace in Fig. 17(*e* ▸).

Substructure determination by single-wavelength anomalous diffraction (SAD) was unsuccessful using data from either visit alone, or using all data combined. However, when using only data from the first 25 images of each data set, a successful substructure solution was obtained (Fig. 17*c* ▸). Unfortunately, the phases were not of good enough quality for subsequent density modification with *SHELXE*. Nonetheless, this demonstrates that careful selection of the data, in particular avoiding inclusion of radiation-damaged data, can be crucial in determining the success of experimental phasing. The correctness of the substructure from SAD phasing was verified by comparison with the SIRAS substructure using the program *phenix.emma* (Adams *et al.*, 2010 ▸).

Anomalous difference maps were calculated with *ANODE* (Thorn & Sheldrick, 2011 ▸), using refined models obtained by running *DIMPLE* on each data set. For all Au data sets two significant anomalous peaks were found. Using all data sets combined gave a stronger anomalous peaks than when only using data from a single beamline visit. However, the strongest anomalous peaks were obtained when using only the first 25 images from each data set (Fig. 17*d* ▸).

While the assumption that all samples are affected by the radiation at the same rate is hard to justify, the effect of individual variation in a population of more than 100 samples is likely to be modest. As such, looking at the population as a whole is reasonable as well as pragmatic, as the entire search space consists of around 10^145^ permutations. It is also worth noting that the completeness of around 90% is an unavoidable feature of some *in situ* data sets, as the samples have preferred orientations with respect to the crystallization plate.

## 7. Discussion and practical recommendations   

Considering the four questions set out earlier.

(i) Is it the case that a larger number of weak observations is equivalent to a smaller number of stronger observations with the same total photon counts? Does the speed of collection matter?
(ii) If very weak data are recorded, are they useful?
(iii) Given a reasonable multiplicity of observations, how is radiation damage detected and how do we decide where to cut the data set?
(iv) Given data from multiple samples, how is it best to combine the data, *i.e.* is it better to combine weak complete sets or stronger partial ones?

Overall, the question of how to use the photons in the absence of radiation damage seems equivocal – by and large the ‘quality’ of the data as assessed by merging statistics is dominated by the total number of scattered photons, at least in the low-dose regime. Of course, radiation damage is rarely absent, so a high-multiplicity/low-dose strategy is a more conservative plan for data collection, provided that a photon-counting detector is used. In general, if a multi-axis goniometer is available and multiple low-dose sweeps are to be recorded, changes in orientation between sweeps (*i.e.* changes in κ or χ) will help to improve the average accuracy of the data. In the absence of any insight into the sample lifetime, recording a full rotation with low flux, say *O*(10^10^) photons per degree, then quadrupling transmission [which will, in the absence of radiation damage and by counting statistics alone, double the 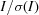 of the data] and repeating until clear signs of radiation damage are seen can be an effective strategy for acquiring a useful data set from a single sample: in the infinite limit the dose deposited before the ‘useful’ data set is roughly one third of the dose of the final set. If the last two sets are used (*i.e.* the ‘useful’ one and the one before with one quarter of the dose) the ‘wasted’ dose (*i.e.* exposure of the sample to X-rays which do not contribute to the final data set) drops to around one twelfth. As shown earlier these weaker data sets can also be useful for confirming the symmetry of the sample, performing molecular replacement or computing difference maps for ligand idenfication. In terms of radiation-damage detection, the *R*_*d*_ statistic (Diederichs, 2006 ▸) can be an effective tool in determining the *presence* of damage though gives little insight into the point at which this damage becomes evident. The *R*_cp_ statistic presented in Section 5.2 overcomes this limitation and may therefore be a useful tool when combined with high-multiplicity/low-dose data collection, and when data are collected *in situ* and the configuration space to explore in terms of cutting back data sets is vast. Finally, the question of combining data from multiple samples and the best data to use remains open. Clearly, assessing isomorphism from effectively complete data sets will be more straightforward than narrow sweeps however the form of data may ultimately be dictated by the mode of data collection *i.e.* *in situ* collection brings geometric limitations. It is however useful to note that combining data from multiple isomorphous samples will almost certainly improve the quality of the final measurements.

As such, the practical recommendations may be summarized as follows.

Collect carefully! In the absence of any insight into the lifetime of your samples, use a low transmission (aiming for *e.g.* 10^11^ photons s^−1^ into a 30 µm beam) and build up from there. Particular care should be taken with microfocus beamlines.
Given a sensible lifetime estimate, record highly multiple data to allow the data set to be truncated later, ideally chang­ing sample orientation between data sets if possible.
Take any detector dead-time into consideration when chosing an exposure time for shutterless data collection – with some detectors such as the Dectris EIGER2 X this is negligible while others (*e.g.* Dectris PILATUS3 X 2M) this can be as much as 24% of the total frame exposure time.
Consider combining data from multiple (isomorphous) samples: if the samples really are representative of the molecule under study and the experiment reproducible, the combined data should be better.
If combining data from multiple samples, analyse the data as they are collected to assess completeness, isomorphism and usefulness of the combined data. For phasing experiments this should include attempts at substructure determination.
Experiment with using different data-processing packages as well as inspecting all available automated processing – the ‘best’ software may be case dependent and some programs may work better than others for your combination of sample, experiment hardware and mode of data collection.

Following these guidelines may increase the computational expense of data analysis and the data storage requirements for archiving. It is worth noting however that low-dose pixel-array data compresses very well (using *gzip* the total storage for a data set is roughly proportional to the total counts in the images) and that careful collection of data may remove the need for collecting from similar samples on a future visit. Of course, the main benefit of the approach presented here is to increase the success rate of X-ray diffraction experiments by limiting the impact of radiation damage, giving the best possible use of your samples and ultimately the best use of photons.

## Supplementary Material

Supporting information file. DOI: 10.1107/S2059798319003528/ba5301sup1.pdf

## Figures and Tables

**Table:** For each data set the diffraction weighted dose was around 0.16 MGy.

	A1	A2	A3	A4
Data collection				
Exposure time (s)	0.02	0.02	0.02	0.02
Ω width (°)	0.15	0.15	0.15	0.15
Transmission (%)	0.42	0.80	0.22	0.42
Number of images	4800	2400	9600	4800
Data processing				
Crystal parameters				
Space group	*I*2_1_3	*I*2_1_3	*I*2_1_3	*I*2_1_3
Unit-cell parameters (Å)	*a* = *b* = *c* = 77.56	*a* = *b* = *c* = 77.58	*a* = *b* = *c* = 77.56	*a* = *b* = *c* = 77.58
Data statistics				
Resolution range (Å)	38.78–1.60 (1.63–1.60)	54.86–1.60 (1.63–1.60)	54.85–1.60 (1.63–1.60)	38.79–1.60 (1.63–1.60)
No. of unique reflections	10428 (528)	10429 (528)	10429 (528)	10431 (529)
Multiplicity	77.0 (76.9)	38.4 (38.4)	154.3 (154.5)	77.0 (76.9)
*R*_merge_	0.191 (3.082)	0.139 (2.225)	0.258 (4.157)	0.201 (3.568)
*R*_meas_	0.192 (3.102)	0.141 (2.255)	0.258 (4.170)	0.202 (3.591)
*R*_p.i.m._	0.022 (0.353)	0.023 (0.363)	0.021 (0.335)	0.023 (0.409)
Completeness (%)	100.0 (100.0)	100.0 (100.0)	100.0 (100.0)	100.0 (100.0)
〈*I*/σ(*I*)〉	18.9 (1.3)	18.0 (1.9)	19.5 (1.1)	17.3 (1.5)
CC_1/2_	1.000 (0.732)	1.000 (0.782)	1.000 (0.739)	1.000 (0.745)
*d*_min_ for CC_1/2_ ≃ 0.5 (Å)	1.45	1.46	1.46	1.47

**Table:** 

	B1	B2	B3	B4
Data collection				
Exposure time (s)	0.02	0.02	0.02	0.02
Ω width (°)	0.15	0.15	0.15	0.15
Transmission (%)	1.52	2.90	0.80	1.52
Number of images	1200	600	2400	1200
Data processing				
Crystal parameters				
Space group	*I*2_1_3	*I*2_1_3	*I*2_1_3	I2_1_3
Unit-cell parameters (Å)	*a* = *b* = *c* = 77.47	*a* = *b* = *c* = 77.47	*a* = *b* = *c* = 77.50	*a* = *b* = *c* = 77.51
Data statistics				
Resolution range (Å)	38.74–1.60 (1.63–1.60)	38.74–1.60 (1.63–1.60)	38.75–1.60 (1.63–1.60)	54.81–1.60 (1.63–1.60)
No. of unique reflections	10379 (526)	10379 (526)	10389 (521)	10390 (521)
Multiplicity	19.4 (19.5)	9.7 (9.8)	38.8 (39.0)	19.4 (19.5)
*R*_merge_	0.142 (1.582)	0.104 (1.186)	0.220 (2.663)	0.142 (1.732)
*R*_meas_	0.146 (1.624)	0.110 (1.252)	0.223 (2.698)	0.146 (1.778)
*R*_p.i.m._	0.033 (0.366)	0.035 (0.399)	0.036 (0.430)	0.033 (0.401)
Completeness (%)	100.0 (100.0)	100.0 (100.0)	100.0 (100.0)	100.0 (100.0)
〈*I*/σ(*I*)〉	12.2 (1.7)	11.2 (1.7)	11.4 (1.1)	12.5 (1.6)
CC_1/2_	0.999 (0.767)	0.999 (0.691)	0.999 (0.701)	0.999 (0.748)
*d*_min_ for CC_1/2_ ≃ 0.5 (Å)	1.45	1.47	1.47	1.48

**Table:** 

	C1	C2	C3	C4
Data collection				
Exposure time (s)	0.02	0.02	0.02	0.02
Ω width (°)	0.15	0.15	0.15	0.15
Transmission (%)	2.90	0.80	1.52	2.90
Number of images	600	2400	1200	600
Data processing				
Crystal parameters				
Space group	*I*2_1_3	*I*2_1_3	*I*2_1_3	*I*2_1_3
Unit-cell parameters (Å)	*a* = *b* = *c* = 77.45	*a* = *b* = *c* = 77.42	*a* = *b* = *c* = 77.46	*a* = *b* = *c* = 77.47
Data statistics				
Resolution range (Å)	38.72–1.60 (1.63–1.60)	54.74–1.60 (1.63–1.60)	38.73–1.60 (1.63–1.60)	54.78–1.60 (1.63–1.60)
No. of unique reflections	10379 (526)	10359 (517)	10389 (521)	10380 (526)
Multiplicity	9.4 (9.5)	38.3 (38.5)	19.1 (19.0)	9.5 (9.6)
*R*_merge_	0.098 (2.400)	0.191 (4.833)	0.129 (3.081)	0.099 (2.511)
*R*_meas_	0.103 (2.539)	0.194 (4.897)	0.132 (3.166)	0.105 (2.654)
*R*_p.i.m._	0.033 (0.823)	0.031 (0.787)	0.030 (0.724)	0.034 (0.855)
Completeness (%)	100.0 (100.0)	100.0 (100.0)	100.0 (100.0)	100.0 (100.0)
〈*I*/σ(*I*)〉	12.4 (1.0)	13.6 (0.8)	14.0 (0.9)	12.5 (0.8)
CC_1/2_	0.999 (0.450)	1.000 (0.474)	1.000 (0.459)	0.999 (0.379)
*d*_min_ for CC_1/2_ ≃ 0.5 (Å)	1.58	1.56	1.56	1.59

**Table 2:** Merging and refinement statistics for thaumatin data sets recorded with transmissions from 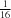
% to 64%, processed to a fixed resolution of 1.6 Å Clearly the weakest of these data are suffering from poor precision in the intensity measurements, which rapidly improve as a greater dose is applied. There is, however, a point of diminishing returns between 1 and 16% where radiation damage becomes a greater factor in data quality than counting statistics, with the optimum data for refinement around 1%, as judged by *R*_free_.

Transmission (%)	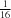	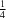	1
Total photons (× 10^9^)	150	610	2440
Total dose (full-beam seconds)	0.09	0.36	1.44
Dose (MGy)	0.012	0.047	0.186
Crystal parameters			
Space group	*P*4_1_2_1_2	*P*4_1_2_1_2	*P*4_1_2_1_2
Unit-cell parameters (Å)	*a* = *b* = 57.82, *c* = 150.13	*a* = *b* = 57.82, *c* = 150.16	*a* = *b* = 57.84, *c* = 150.21
Data statistics			
Resolution range (Å)	53.96–1.60 (1.63–1.60)	50.05–1.60 (1.63–1.60)	150.21–1.60 (1.63–1.60)
No. of unique reflections	34720 (1677)	34696 (1670)	34726 (1662)
Multiplicity	24.2 (23.8)	24.1 (23.6)	24.0 (23.5)
*R*_merge_	0.411 (4.412)	0.223 (1.840)	0.142 (0.839)
*R*_meas_	0.420 (4.509)	0.228 (1.880)	0.145 (0.858)
*R*_p.i.m._	0.085 (0.921)	0.046 (0.385)	0.029 (0.176)
Completeness (%)	100.0 (99.8)	100.0 (99.6)	100.0 (99.1)
〈*I*/σ(*I*)〉	5.8 (0.8)	9.9 (1.8)	14.4 (3.4)
CC_1/2_	0.996 (0.493)	0.998 (0.837)	0.999 (0.954)
*R*_work_	0.1777	0.1650	0.1605
*R*_free_	0.2108	0.1983	0.1916
Transmission (%)	4	16	64
Total photons (× 10^12^)	9.84	39.1	157.0
Total dose (full-beam seconds)	5.79	23.00	92.33
Dose (MGy)	0.748	2.971	11.926
Crystal parameters			
Space group	*P*4_1_2_1_2	*P*4_1_2_1_2	*P*4_1_2_1_2
Unit-cell parameters (Å)	*a* = *b* = 57.87, *c* = 150.27	*a* = *b* = 57.92, *c* = 150.41	*a* = *b* = 57.97, *c* = 150.53
Data statistics			
Resolution range (Å)	150.27–1.60 (1.63–1.60)	75.20–1.60 (1.63–1.60)	57.97–1.60 (1.63–1.60)
No. of unique reflections	34761 (1668)	34850 (1674)	34971 (1662)
Multiplicity	23.9 (23.5)	23.8 (23.2)	23.6 (22.6)
*R*_merge_	0.118 (0.495)	0.117 (0.435)	0.181 (1.345)
*R*_meas_	0.121 (0.506)	0.119 (0.445)	0.185 (1.377)
*R*_p.i.m._	0.024 (0.104)	0.024 (0.092)	0.038 (0.291)
Completeness (%)	100.0 (99.7)	100.0 (100.0)	100.0 (97.4)
〈*I*/σ(*I*)〉	16.8 (4.6)	16.6 (3.7)	11.4 (1.6)
CC_1/2_	0.999 (0.981)	0.999 (0.985)	0.998 (0.912)
*R*_work_	0.1608	0.1676	0.1815
*R*_free_	0.1920	0.2010	0.2208

**Table 3:** Point-group symmetry-analysis scores for individual rotational symmetry operations for a low-dose data set from CDK2

Likelihood	*Z*_cc_	CC	*N*	*R*_meas_	Score	Symmetry operation
0.900	8.23	0.82	85649	0.231		Identity
0.857	7.17	0.72	84564	0.303	**	Twofold *l*
0.909	7.91	0.79	76905	0.266	***	Twofold *k*
0.908	7.70	0.77	77473	0.277	***	Twofold *h*
0.057	0.66	0.07	85634	0.884		Twofold
0.058	1.13	0.11	83185	0.851		Twofold
0.057	0.83	0.08	152231	0.818		Fourfold *l*

**Table 4:** Merging statistics for data derived from the first insulin crystal, with digital transmission applied The data are indexed by the transmission factor from 1 to 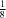 and the total rotation included (*i.e.* all 720°, first 360°, 180° and 90° of the data). Data sets in each row are in principle comparable, as the product of the rotation and transmission factor is constant.

	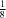 − 720°	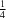 − 360°	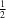 − 180°	1 − 90°
Crystal parameters				
Space group	*I*2_1_3	*I*2_1_3	*I*2_1_3	*I*2_1_3
Unit-cell parameters (Å)	*a* = *b* = *c* = 78.13	*a* = *b* = *c* = 78.12	*a* = *b* = *c* = 78.12	*a* = *b* = *c* = 78.12
Data statistics				
Resolution range (Å)	39.07–1.40 (1.42–1.40)	55.24–1.40 (1.42–1.40)	39.06–1.40 (1.42–1.40)	39.06–1.40 (1.42–1.40)
No. of unique reflections	15814 (803)	15789 (777)	15788 (777)	15788 (777)
Multiplicity	75.2 (64.1)	37.6 (32.3)	18.8 (16.1)	9.4 (8.0)
*R*_merge_	0.095 (2.555)	0.074 (1.672)	0.059 (1.105)	0.049 (0.777)
*R*_meas_	0.096 (2.576)	0.075 (1.699)	0.060 (1.141)	0.052 (0.830)
*R*_p.i.m._	0.011 (0.321)	0.012 (0.298)	0.014 (0.283)	0.017 (0.290)
Completeness (%)	100.0 (100.0)	100.0 (100.0)	100.0 (100.0)	100.0 (100.0)
〈*I*/σ(*I*)〉	27.7 (1.8)	25.7 (2.0)	23.4 (2.1)	20.2 (2.1)
CC_1/2_	1.000 (0.790)	1.000 (0.797)	0.999 (0.818)	0.998 (0.813)
	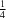 − 720°	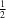 − 360°	1 − 180°	
Crystal parameters				
Space group	*I*2_1_3	*I*2_1_3	*I*2_1_3	
Unit-cell parameters (Å)	*a* = *b* = *c* = 78.13	*a* = *b* = *c* = 78.12	*a* = *b* = *c* = 78.11	
Data statistics				
Resolution range (Å)	39.07–1.40 (1.42–1.40)	55.24–1.40 (1.42–1.40)	55.23–1.40 (1.42–1.40)	
No. of unique reflections	15814 (803)	15789 (777)	15781 (799)	
Multiplicity	75.2 (64.0)	37.6 (32.3)	18.8 (16.2)	
*R*_merge_	0.077 (1.790)	0.061 (1.176)	0.051 (0.777)	
*R*_meas_	0.077 (1.804)	0.062 (1.195)	0.052 (0.802)	
*R*_p.i.m._	0.009 (0.225)	0.010 (0.210)	0.012 (0.199)	
Completeness (%)	100.0 (100.0)	100.0 (100.0)	100.0 (100.0)	
〈*I*/σ(*I*)〉	35.2 (2.7)	32.2 (2.9)	28.7 (3.1)	
CC_1/2_	1.000 (0.876)	1.000 (0.880)	0.999 (0.898)	
	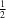 − 720°	1 − 360°		
Crystal parameters				
Space group	*I*2_1_3	*I*2_1_3		
Unit-cell parameters (Å)	*a* = *b* = *c* = 78.13	*a* = *b* = *c* = 78.12		
Data statistics				
Resolution range (Å)	55.25–1.40 (1.42–1.40)	55.24–1.40 (1.42–1.40)		
No. of unique reflections	15815 (803)	15789 (777)		
Multiplicity	75.1 (63.8)	37.5 (32.2)		
*R*_merge_	0.064 (1.264)	0.053 (0.834)		
*R*_meas_	0.064 (1.274)	0.054 (0.847)		
*R*_p.i.m._	0.007 (0.159)	0.009 (0.149)		
Completeness (%)	100.0 (100.0)	100.0 (100.0)		
〈*I*/σ(*I*)〉	43.6 (4.0)	39.3 (4.2)		
CC_1/2_	1.000 (0.928)	0.999 (0.943)		
	1 − 720°			
Crystal parameters				
Space group	*I*2_1_3			
Unit-cell parameters (Å)	*a* = *b* = *c* = 78.13			
Data statistics				
Resolution range (Å)	39.06–1.40 (1.42–1.40)			
No. of unique reflections	15814 (803)			
Multiplicity	75.0 (63.4)			
*R*_merge_	0.055 (0.896)			
*R*_meas_	0.056 (0.903)			
*R*_p.i.m._	0.006 (0.113)			
Completeness (%)	100.0 (100.0)			
〈*I*/σ(*I*)〉	53.2 (5.6)			
CC_1/2_	1.000 (0.968)			

**Table:** 

No. of data sets	1	2
Crystal parameters		
Space group	*P*6_1_22	*P*6_1_22
Unit-cell parameters (Å)	*a* = *b* = 92.36, *c* = 127.72	*a* = *b* = 92.36, *c* = 127.72
Data statistics		
Resolution range (Å)	67.79–1.55 (1.58–1.55)	67.79–1.55 (1.58–1.55)
No. of unique reflections	46567 (2200)	46572 (2195)
Multiplicity	74.9 (66.8)	149.7 (133.4)
*R*_merge_	0.214 (4.511)	0.216 (4.520)
*R*_meas_	0.216 (4.545)	0.217 (4.537)
*R*_p.i.m._	0.024 (0.547)	0.017 (0.387)
Completeness (%)	98.6 (95.2)	98.6 (95.3)
〈*I*/σ(*I*)〉	15.3 (0.9)	21.5 (1.3)
CC_1/2_	1.000 (0.529)	1.000 (0.687)
Resolution range (Å)	60.01–1.55 (1.59–1.55)	60.01–1.55 (1.59–1.55)
No. of reflections		
Total	46461 (3276)	46486 (3272)
Working set	44168 (3083)	44195 (3079)
Free set	2293 (193)	2291 (193)
*R*_work_	0.142 (0.308)	0.139 (0.254)
*R*_free_	0.176 (0.353)	0.172 (0.283)
No. of non-H atoms	2801	2801
R.m.s. deviations from ideal		
Bond lengths (Å)	0.008	0.008
Bond angles (°)	1.261	1.234

**Table:** 

No. of data sets	4	8
Crystal parameters		
Space group	*P*6_1_22	*P*6_1_22
Unit-cell parameters (Å)	*a* = *b* = 92.37, *c* = 127.73	*a* = *b* = 92.38, *c* = 127.74
Data statistics		
Resolution range (Å)	127.73–1.55 (1.58–1.55)	80.00–1.55 (1.58–1.55)
No. of unique reflections	46586 (2198)	46612 (2205)
Multiplicity	299.5 (266.5)	598.8 (530.9)
*R*_merge_	0.220 (4.665)	0.229 (4.929)
*R*_meas_	0.221 (4.673)	0.229 (4.934)
*R*_p.i.m._	0.013 (0.282)	0.009 (0.211)
Completeness (%)	98.6 (95.2)	98.6 (95.3)
〈*I*/σ(*I*)〉	30.2 (1.8)	41.2 (2.4)
CC_1/2_	1.000 (0.812)	1.000 (0.896)
Resolution range (Å)	60.01–1.55 (1.59–1.55)	60.01–1.55 (1.59–1.55)
No. of reflections		
Total	46499 (3274)	46520 (3280)
Working set	44205 (3083)	44224 (3089)
Free set	2294 (191)	2296 (191)
*R*_work_	0.137 (0.215)	0.138 (0.218)
*R*_free_	0.169 (0.246)	0.170 (0.275)
No. of non-H atoms	2801	2801
R.m.s. deviations from ideal		
Bond lengths (Å)	0.007	0.008
Bond angles (°)	1.229	1.232

**Table 6:** Merging statistics for CDK2 for the full data set (four full rotations) one half and one quarter, the last as recommended by interpretation of *R*_cp_ All data are processed to a fixed resolution limit of 1.3 Å to enable straightforward comparison. Though the *R*_p.i.m._ in the outer resolution shell improves slightly in the full data set, it is a long way short of the improvement which could be expected from the fourfold increase in multiplicity.

Subset	1-9600	1-4800	1-2400
Crystal parameters			
Space group	*P*2_1_2_1_2_1_	*P*2_1_2_1_2_1_	*P*2_1_2_1_2_1_
Unit-cell *a* (Å)	53.43	53.42	53.40
Unit-cell *b* (Å)	72.11	72.08	72.06
Unit-cell *c* (Å)	72.60	72.60	72.59
Data statistics			
Resolution range (Å)	72.11–1.30 (1.32–1.30)	72.60–1.30 (1.32–1.30)	72.59–1.30 (1.32–1.30)
No. of unique reflections	69717 (3410)	69657 (3412)	69610 (3395)
Multiplicity	49.3 (48.4)	24.6 (24.3)	12.3 (12.1)
*R*_merge_	0.056 (0.581)	0.045 (0.416)	0.040 (0.347)
*R*_meas_	0.057 (0.588)	0.046 (0.425)	0.041 (0.362)
*R*_p.i.m._	0.008 (0.084)	0.009 (0.085)	0.012 (0.103)
Completeness (%)	100.0 (99.4)	100.0 (99.9)	100.0 (99.4)
〈*I*/σ(*I*)〉	39.2 (5.6)	34.3 (5.0)	26.1 (3.9)
CC_1/2_	1.000 (0.984)	1.000 (0.978)	1.000 (0.965)

**Table 7:** Merging statistics for four 360° data sets from cubic insulin. Each data set was recorded with a low transmission to reduce the impact of radiation damage A fixed resolution limit of 1.4 Å was used for side-by-side comparisons.

Crystal (×360°)	1	2	3	4
Crystal parameters				
Space group	*I*2_1_3	*I*2_1_3	*I*2_1_3	*I*2_1_3
Unit-cell parameters (Å)	*a* = *b* = *c* = 78.12	*a* = *b* = *c* = 78.07	*a* = *b* = *c* = 78.11	*a* = *b* = *c* = 78.04
Data statistics				
Resolution range (Å)	55.24–1.40 (1.42–1.40)	39.03–1.40 (1.42–1.40)	39.05–1.40 (1.42–1.40)	39.02–1.40 (1.42–1.40)
No. of unique reflections	15789 (777)	15748 (767)	15788 (777)	15740 (787)
Multiplicity	37.5 (32.2)	37.6 (32.4)	37.6 (31.9)	37.6 (32.4)
*R*_merge_	0.053 (0.834)	0.055 (0.782)	0.055 (0.855)	0.061 (0.782)
*R*_meas_	0.054 (0.847)	0.056 (0.794)	0.056 (0.869)	0.062 (0.794)
*R*_p.i.m._	0.009 (0.149)	0.009 (0.139)	0.009 (0.153)	0.010 (0.139)
Completeness (%)	100.0 (100.0)	100.0 (100.0)	100.0 (100.0)	100.0 (100.0)
〈*I*/σ(*I*)〉	39.3 (4.2)	38.3 (4.7)	37.0 (4.1)	35.6 (4.7)
CC_1/2_	0.999 (0.943)	1.000 (0.943)	0.999 (0.938)	0.999 (0.939)

**Table 9:** Merging statistics from accumulating data from 360° of samples 1, 1 + 2, 1 + 2 + 3 and 1 + 2 + 3 + 4 As expected, including carefully measured data from multiple samples makes for a clear improvement in 〈*I*/σ(*I*)〉 and phasing success (Fig. 15 ▸).

Crystals (×360°)	1	1 + 2	1 + 2 + 3	1 + 2 + 3 + 4
Crystal parameters				
Space group	*I*2_1_3	*I*2_1_3	*I*2_1_3	*I*2_1_3
Unit-cell parameters (Å)	*a* = *b* = *c* = 78.12	*a* = *b* = *c* = 78.09	*a* = *b* = *c* = 78.10	*a* = *b* = *c* = 78.08
Data statistics				
Resolution range (Å)	55.24–1.40 (1.42–1.40)	55.22–1.40 (1.42–1.40)	39.05–1.40 (1.42–1.40)	55.21–1.40 (1.42–1.40)
No. of unique reflections	15789 (777)	15781 (799)	15780 (799)	15781 (799)
Multiplicity	37.5 (32.2)	75.1 (63.7)	112.6 (95.9)	150.1 (126.7)
*R*_merge_	0.053 (0.834)	0.054 (0.811)	0.055 (0.828)	0.058 (0.820)
*R*_meas_	0.054 (0.847)	0.055 (0.817)	0.055 (0.832)	0.058 (0.823)
*R*_p.i.m._	0.009 (0.149)	0.006 (0.102)	0.005 (0.085)	0.005 (0.073)
Completeness (%)	100.0 (100.0)	100.0 (100.0)	100.0 (100.0)	100.0 (100.0)
〈*I*/σ(*I*)〉	39.3 (4.2)	54.8 (6.2)	65.9 (7.5)	72.9 (8.9)
CC_1/2_	0.999 (0.943)	1.000 (0.969)	0.999 (0.979)	1.000 (0.983)

**Table 8:** Reproduced statistics from Table 7 ▸, with combined half data sets from samples 1 + 2 and 3 + 4 and quarter data sets of 1 + 2 + 3 + 4, showing comparable statistics in all cases to the resolution limit of 1.4 Å

Crystal	1, 360°	2, 360°	1 + 2, 180°	1 + 2 + 3 + 4, 90°
Crystal parameters				
Space group	*I*2_1_3	*I*2_1_3	*I*2_1_3	*I*2_1_3
Unit-cell parameters (Å)	*a* = *b* = *c* = 78.12	*a* = *b* = *c* = 78.07	*a* = *b* = *c* = 78.09	*a* = *b* = *c* = 78.08
Data statistics				
Resolution range (Å)	55.24–1.40 (1.42–1.40)	39.03–1.40 (1.42–1.40)	39.04–1.40 (1.42–1.40)	55.21–1.40 (1.42–1.40)
No. of unique reflections	15789 (777)	15748 (767)	15780 (799)	15781 (799)
Multiplicity	37.5 (32.2)	37.6 (32.4)	37.5 (31.7)	37.4 (31.4)
*R*_merge_	0.053 (0.834)	0.055 (0.782)	0.053 (0.774)	0.056 (0.804)
*R*_meas_	0.054 (0.847)	0.056 (0.794)	0.053 (0.787)	0.056 (0.817)
*R*_p.i.m._	0.009 (0.149)	0.009 (0.139)	0.009 (0.139)	0.009 (0.145)
Completeness (%)	100.0 (100.0)	100.0 (100.0)	100.0 (100.0)	100.0 (100.0)
〈*I*/σ(*I*)〉	39.3 (4.2)	38.3 (4.7)	39.9 (4.5)	37.3 (4.4)
CC_1/2_	0.999 (0.943)	1.000 (0.943)	0.999 (0.945)	0.999 (0.943)
Crystal	3, 360°	4, 360°	3 + 4, 180°	
Crystal parameters				
Space group	*I*2_1_3	*I*2_1_3	*I*2_1_3	
Unit-cell parameters (Å)	*a* = *b* = *c* = 78.11	*a* = *b* = *c* = 78.04	*a* = *b* = *c* = 78.07	
Data statistics				
Resolution range (Å)	39.05–1.40 (1.42–1.40)	39.02–1.40 (1.42–1.40)	55.21–1.40 (1.42–1.40)	
No. of unique reflections	15788 (777)	15740 (787)	15749 (767)	
Multiplicity	37.6 (31.9)	37.6 (32.4)	37.5 (32.0)	
*R*_merge_	0.055 (0.855)	0.061 (0.782)	0.059 (0.801)	
*R*_meas_	0.056 (0.869)	0.062 (0.794)	0.059 (0.813)	
*R*_p.i.m._	0.009 (0.153)	0.010 (0.139)	0.010 (0.143)	
Completeness (%)	100.0 (100.0)	100.0 (100.0)	100.0 (100.0)	
〈*I*/σ(*I*)〉	37.0 (4.1)	35.6 (4.7)	35.5 (4.6)	
CC_1/2_	0.999 (0.938)	0.999 (0.939)	1.000 (0.938)	

**Table 10:** Merging statistics for native and Au-derivative data sets of proteinase K. Statistics are reported for Au-derivative data sets collected separately across two beamline visits, with all data combined, and using only the first 25 images from each data set.

	Native	Au (visit 1)	Au (visit 2)
Number of data sets	75	58	75
Crystal parameters			
Space group	*P*4_3_2_1_2	*P*4_3_2_1_2	*P*4_3_2_1_2
Unit-cell parameters (Å)	*a* = *b* = 68.26, *c* = 103.49	*a* = *b* = 68.45, *c* = 103.83	*a* = *b* = 68.50, *c* = 104.08
Data statistics			
Resolution range (Å)	68.33–1.58 (1.64–1.58)	68.53–1.44 (1.49–1.44)	68.55–1.69 (1.75–1.69)
No. of reflections	317754 (354)	528243 (5083)	518792 (2261)
Multiplicity	10.3 (1.7)	12.7 (5.8)	19.7 (3.3)
*R*_merge_	0.281 (1.694)	0.559 (3.772)	1.407 (1.918)
*R*_meas_	0.295 (2.091)	0.583 (4.061)	1.443 (2.137)
*R*_p.i.m._	0.085 (1.190)	0.161 (1.371)	0.314 (0.843)
Completeness (%)	89.8 (6.3)	92.0 (19.6)	92.5 (24.7)
〈*I*/σ(*I*)〉	9.4 (0.4)	6.7 (0.3)	5.5 (0.5)
CC_1/2_	0.983 (0.358)	0.970 (0.117)	0.918 (0.043)
	Au (all)	Au (1–25)	
Number of data sets	133	133	
Crystal parameters			
Space group	*P*4_3_2_1_2	*P*4_3_2_1_2	
Unit-cell parameters (Å)	*a* = *b* = 68.45, *c* = 103.83	*a* = *b* = 68.45, *c* = 103.83	
Data statistics			
Resolution range (Å)	68.53–1.49 (1.55–1.49)	68.54–1.40 (1.45–1.40)	
No. of reflections	1200900 (1329)	1012029 (2437)	
Multiplicity	31.9 (1.6)	22.3 (2.7)	
*R*_merge_	0.922 (1.269)	0.583 (13.525)	
*R*_meas_	0.937 (1.631)	0.597 (15.893)	
*R*_p.i.m._	0.163 (1.007)	0.126 (7.640)	
Completeness (%)	92.1 (20.5)	92.0 (18.5)	
〈*I*/σ(*I*)〉	7.0 (0.3)	7.6 (0.1)	
CC_1/2_	0.969 (-0.036)	0.971 (-0.029)	

## Footnotes

1 That is real numbers greater than or equal to 0 but strictly less than 1.

2 The number of trials used were to improve the resolution of the histogram, rather than increasing the likelihood of success.
