# Supplementary material for: How best to use photons
Source: Acta Crystallogr D Struct Biol. 2019 Mar 19;75(Pt 3):242–61. doi: 10.1107/S2059798319003528 (PMC6450062; doi:10.1107/S2059798319003528)
Supplement: Supplementary file 1 [file d-75-00242-sup1.pdf]

STRUCTURAL BIOLOGY

Volume 75 (2019)

Supporting information for article:

How best to use photons

Graeme Winter, Richard J. Gildea, Neil Paterson, John Beale, Markus Gerstel, Danny Axford, Melanie Vollmar, Katherine E. McAuley, Robin L. Owen, Ralf Flaig, Alun W. Ashton and David R. Hall

Appendix A
Preparation of Small Cubic Insulin Crystals

Bovine insulin powder (Sigma-Aldrich, I5500), was solubilised to a final concentration of 25 mg/mL in double distilled water. This solution was filtered through a 0.2 μm centrifugal filter (EMD Millipore), flash-cooled in liquid nitrogen and stored at -80 °C until used. Initial cubic insulin crystals were grown in CrystalQuick, 96-well sitting drop plates (Greiner) by mixing the insulin solution and the crystallisation solution, 0.1 M BIS-TRIS propane pH 7.5, 0.1 M pH 7.5, 0.1 M potassium iodide and 22 % w/v PEG 3350 (all procured from Sigma-Aldrich), in a 1:1 ratio to total drop volume of 400 nL. After approximately 24 hr, all crystals from five drops were crushed and pooled in 250 μL of crystallisation buffer cooled on ice. A seed stock was created by adding five 1 mm glass milled beads (Fisher Scientific) to the crushed crystals, this solution was then vortexed and cooled on ice for 30 s concurrently three times. A 100x dilution of this seed stock in more crystallisation buffer was created and could be stored at -20 °C until required. The cubic insulin crystals used for the I24 data collection were produced by mixing this 100x seed stock dilution, crystallisation buffer and 25 mg/mL insulin solution in a 1:2:3 ratio to a total volume of 300 nL. Approximately 50 x 50 x 50 μm crystals were harvested using 50 μm MicoMounts (MiTeGen) after 24 hr, cryo-protected in crystallisation buffer with 25 % v/v ethylene

glycol (Sigma-Aldrich), and flash cooled in liquid nitrogen.

Appendix B
Scripts

B.1. Insulin S-SAD with SHELX C/D/E

The following script was used to attempt phasing from the disulphides present in insulin, derived from the scripts presented in the SHELX C/D/E documentation:

```
shelxc ins << eof
spag I213
cell 78.12816 78.12816 78.12816 90.0        90.0        90.0
find 6
dsul 3
mind -3.5
sad ../DataFiles/AUTOMATIC_DEFAULT_scaled_unmerged.sca
eof

shelxd ins_fa
shelxe ins ins_fa -s0.65 -m20 -h
shelxe ins ins_fa -s0.65 -m20 -h -i
```

Appendix C
Cubic Insulin / Semi-Synthetic Exploration

C.1. Evidence of Mild Radiation Damage in Original Data

The full 720° data sets were processed with `xia2` / `DIALS` using AIMLESS (Evans & Murshudov, 2013) for scaling. R_d plots (Diederichs, 2006) (Figure S1) show signs of modest damage on the timescale of the complete data set though as may be seen from Tables S1 to S4 the overall R_{pim} are still somewhat better than any of the subsets.

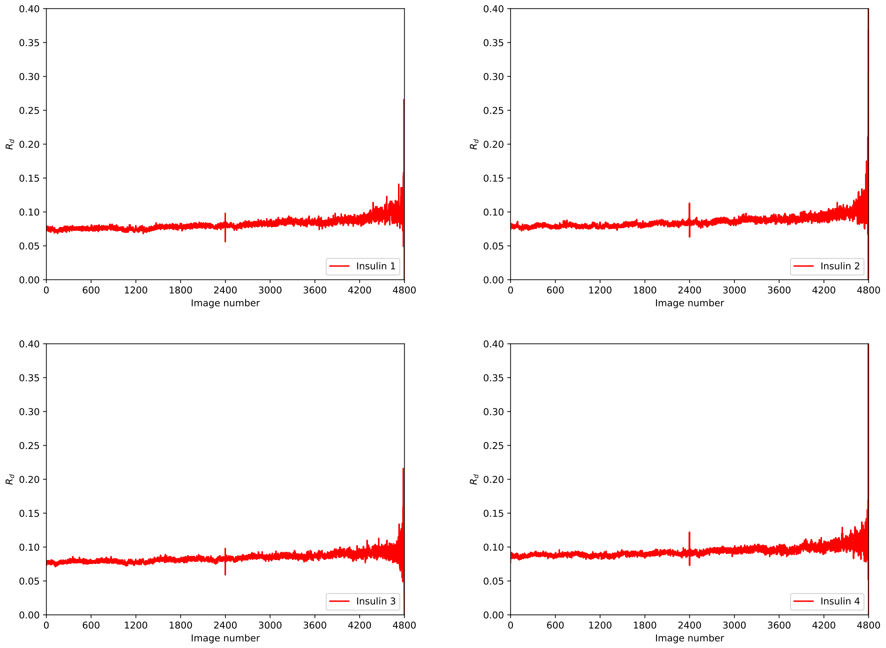

Fig. S1. R_d plots for the full 720° data sets for each of the four cubic insulin data sets referenced in Section 3.3.

C.2. Command Line for Digital Attenuation

The command-line tools used to generate the digitally attenuated data in Section‘3.3.1 are included in recent `DIALS` distributions. and currently work on PILATUS CBF images. The tool `dev.dials.scale_down_images` takes the original sequence of images as a filename template (using `printf` syntax), as well as a template to use for the output images, the first and last image number and the scale factor to apply, as:

```
dev.dials.scale_down_images ../original/INS1_2_%04d.cbf \
../0.5-scale/SCALED_INS1_2_%04d.cbf 1 4800 0.5
```

The file headers are copied verbatim so maintain all original metadata, while the image which follows will be appropriately scaled. The tool is not intended for routine data processing but helpful for methods development.

C.3. Tables of Merging Statistics for Cubic Insulin Crystals

Each of the data sets described in Section 3.3 were processed with `xia2` / `DIALS`, as full and partial data sets as well as being digitally attenuated with factors $\frac{1}{2}$, $\frac{1}{4}$ and $\frac{1}{8}$. The tables below show the full merging statistics for each permutation, processed to 1.4 Å for the sake of simple comparison. Within each *row* the data sets are in principle comparable (modulo the modest radiation damage described above) as the total scattered photons are approximately equivalent, while each *column* shows the effect of attenuating the data whilst maintaining the same total rotation.

	$\frac{1}{8} - 720°$	$\frac{1}{4} - 360°$	$\frac{1}{2} - 180°$	$1 - 90°$
Crystal parameters				
Space group	$I2_13$	$I2_13$	$I2_13$	$I2_13$
Unit-cell parameters (Å)	$a = b = c = 78.13$	$a = b = c = 78.12$	$a = b = c = 78.12$	$a = b = c = 78.12$
Data statistics				
Resolution range (Å)	39.07-1.40 (1.42-1.40)	55.24-1.40 (1.42-1.40)	39.06-1.40 (1.42-1.40)	39.06-1.40 (1.42-1.40)
No. of unique reflections	15814 (803)	15789 (777)	15788 (777)	15788 (777)
Multiplicity	75.2 (64.1)	37.6 (32.3)	18.8 (16.1)	9.4 (8.0)
R_{merge}	0.095 (2.555)	0.074 (1.672)	0.059 (1.105)	0.049 (0.777)
R_{meas}	0.096 (2.576)	0.075 (1.699)	0.060 (1.141)	0.052 (0.830)
R_{pim}	0.011 (0.321)	0.012 (0.298)	0.014 (0.283)	0.017 (0.290)
Completeness (%)	100.0 (100.0)	100.0 (100.0)	100.0 (100.0)	100.0 (100.0)
$< I/\sigma(I) >$	27.7 (1.8)	25.7 (2.0)	23.4 (2.1)	20.2 (2.1)
$CC_{\frac{1}{2}}$	1.000 (0.790)	1.000 (0.797)	0.999 (0.818)	0.998 (0.813)
	$\frac{1}{4} - 720°$	$\frac{1}{2} - 360°$	$1 - 180°$	
Crystal parameters				
Space group	$I2_13$	$I2_13$	$I2_13$	
Unit-cell parameters (Å)	$a = b = c = 78.13$	$a = b = c = 78.12$	$a = b = c = 78.11$	
Data statistics				
Resolution range (Å)	39.07-1.40 (1.42-1.40)	55.24-1.40 (1.42-1.40)	55.23-1.40 (1.42-1.40)	
No. of unique reflections	15814 (803)	15789 (777)	15781 (799)	
Multiplicity	75.2 (64.0)	37.6 (32.3)	18.8 (16.2)	
R_{merge}	0.077 (1.790)	0.061 (1.176)	0.051 (0.777)	
R_{meas}	0.077 (1.804)	0.062 (1.195)	0.052 (0.802)	
R_{pim}	0.009 (0.225)	0.010 (0.210)	0.012 (0.199)	
Completeness (%)	100.0 (100.0)	100.0 (100.0)	100.0 (100.0)	
$< I/\sigma(I) >$	35.2 (2.7)	32.2 (2.9)	28.7 (3.1)	
$CC_{\frac{1}{2}}$	1.000 (0.876)	1.000 (0.880)	0.999 (0.898)	
	$\frac{1}{2} - 720°$	$1 - 360°$		
Crystal parameters				
Space group	$I2_13$	$I2_13$		
Unit-cell parameters (Å)	$a = b = c = 78.13$	$a = b = c = 78.12$		
Data statistics				
Resolution range (Å)	55.25-1.40 (1.42-1.40)	55.24-1.40 (1.42-1.40)		
No. of unique reflections	15815 (803)	15789 (777)		
Multiplicity	75.1 (63.8)	37.5 (32.2)		
R_{merge}	0.064 (1.264)	0.053 (0.834)		
R_{meas}	0.064 (1.274)	0.054 (0.847)		
R_{pim}	0.007 (0.159)	0.009 (0.149)		
Completeness (%)	100.0 (100.0)	100.0 (100.0)		
$< I/\sigma(I) >$	43.6 (4.0)	39.3 (4.2)		
$CC_{\frac{1}{2}}$	1.000 (0.928)	0.999 (0.943)		
	$1 - 720°$			
Crystal parameters				
Space group	$I2_13$			
Unit-cell parameters (Å)	$a = b = c = 78.13$			
Data statistics				
Resolution range (Å)	39.06-1.40 (1.42-1.40)			
No. of unique reflections	15814 (803)			
Multiplicity	75.0 (63.4)			
R_{merge}	0.055 (0.896)			
R_{meas}	0.056 (0.903)			
R_{pim}	0.006 (0.113)			
Completeness (%)	100.0 (100.0)			
$< I/\sigma(I) >$	53.2 (5.6)			
$CC_{\frac{1}{2}}$	1.000 (0.968)			

Table S1. *Merging statistics for all combinations of subsets of data and digital transmissions for insulin crystal 1*

	$\frac{1}{8} - 720^\circ$	$\frac{1}{4} - 360^\circ$	$\frac{1}{2} - 180^\circ$	$1 - 90^\circ$
Crystal parameters				
Space group	$I2_13$	$I2_13$	$I2_13$	$I2_13$
Unit-cell parameters (Å)	$a = b = c = 78.08$	$a = b = c = 78.07$	$a = b = c = 78.07$	$a = b = c = 78.06$
Data statistics				
Resolution range (Å)	55.21-1.40 (1.42-1.40)	39.04-1.40 (1.42-1.40)	39.03-1.40 (1.42-1.40)	39.03-1.40 (1.42-1.40)
No. of unique reflections	15781 (799)	15748 (767)	15748 (767)	15748 (767)
Multiplicity	75.3 (63.6)	37.6 (32.5)	18.8 (16.2)	9.4 (8.0)
R_{merge}	0.098 (2.414)	0.075 (1.572)	0.060 (1.056)	0.051 (0.767)
R_{meas}	0.099 (2.434)	0.076 (1.596)	0.062 (1.090)	0.053 (0.820)
R_{pim}	0.011 (0.304)	0.012 (0.279)	0.014 (0.270)	0.017 (0.286)
Completeness (%)	100.0 (100.0)	100.0 (100.0)	100.0 (100.0)	100.0 (100.0)
$< I/\sigma(I) >$	27.4 (1.9)	25.4 (2.2)	23.0 (2.3)	19.7 (2.2)
$CC_{\frac{1}{2}}$	1.000 (0.795)	0.999 (0.811)	0.997 (0.812)	0.999 (0.771)

	$\frac{1}{4} - 720^\circ$	$\frac{1}{2} - 360^\circ$	$1 - 180^\circ$
Crystal parameters			
Space group	$I2_13$	$I2_13$	$I2_13$
Unit-cell parameters (Å)	$a = b = c = 78.08$	$a = b = c = 78.07$	$a = b = c = 78.07$
Data statistics			
Resolution range (Å)	55.21-1.40 (1.42-1.40)	39.04-1.40 (1.42-1.40)	39.03-1.40 (1.42-1.40)
No. of unique reflections	15749 (767)	15748 (767)	15748 (767)
Multiplicity	75.3 (65.0)	37.6 (32.5)	18.8 (16.2)
R_{merge}	0.078 (1.652)	0.063 (1.102)	0.052 (0.747)
R_{meas}	0.079 (1.665)	0.064 (1.119)	0.054 (0.772)
R_{pim}	0.009 (0.206)	0.010 (0.196)	0.012 (0.191)
Completeness (%)	100.0 (100.0)	100.0 (100.0)	100.0 (100.0)
$< I/\sigma(I) >$	34.8 (3.0)	31.6 (3.2)	28.2 (3.4)
$CC_{\frac{1}{2}}$	1.000 (0.882)	0.999 (0.894)	0.997 (0.894)

	$\frac{1}{2} - 720^\circ$	$1 - 360^\circ$
Crystal parameters		
Space group	$I2_13$	$I2_13$
Unit-cell parameters (Å)	$a = b = c = 78.08$	$a = b = c = 78.07$
Data statistics		
Resolution range (Å)	55.21-1.40 (1.42-1.40)	39.03-1.40 (1.42-1.40)
No. of unique reflections	15749 (767)	15748 (767)
Multiplicity	75.3 (65.0)	37.6 (32.4)
R_{merge}	0.066 (1.165)	0.055 (0.782)
R_{meas}	0.066 (1.174)	0.056 (0.794)
R_{pim}	0.008 (0.145)	0.009 (0.139)
Completeness (%)	100.0 (100.0)	100.0 (100.0)
$< I/\sigma(I) >$	43.1 (4.5)	38.3 (4.7)
$CC_{\frac{1}{2}}$	1.000 (0.935)	1.000 (0.943)

	$1 - 720^\circ$
Crystal parameters	
Space group	$I2_13$
Unit-cell parameters (Å)	$a = b = c = 78.08$
Data statistics	
Resolution range (Å)	39.04-1.40 (1.42-1.40)
No. of unique reflections	15748 (767)
Multiplicity	75.3 (64.9)
R_{merge}	0.058 (0.822)
R_{meas}	0.058 (0.828)
R_{pim}	0.007 (0.102)
Completeness (%)	100.0 (100.0)
$< I/\sigma(I) >$	52.0 (6.4)
$CC_{\frac{1}{2}}$	1.000 (0.964)

Table S2. *Merging statistics for all combinations of subsets of data and digital transmissions for insulin crystal 2*

	$\frac{1}{8} - 720^\circ$	$\frac{1}{4} - 360^\circ$	$\frac{1}{2} - 180^\circ$	$1 - 90^\circ$
Crystal parameters				
Space group	$I2_13$	$I2_13$	$I2_13$	$I2_13$
Unit-cell parameters (Å)	$a = b = c = 78.12$	$a = b = c = 78.11$	$a = b = c = 78.11$	$a = b = c = 78.10$
Data statistics				
Resolution range (Å)	55.24-1.40 (1.42-1.40)	39.06-1.40 (1.42-1.40)	55.23-1.40 (1.42-1.40)	39.05-1.40 (1.42-1.40)
No. of unique reflections	15789 (777)	15788 (777)	15789 (777)	15780 (799)
Multiplicity	75.3 (64.3)	37.6 (32.0)	18.8 (16.0)	9.4 (8.0)
R_{merge}	0.097 (2.637)	0.075 (1.739)	0.060 (1.163)	0.053 (0.833)
R_{meas}	0.097 (2.658)	0.076 (1.766)	0.062 (1.202)	0.056 (0.891)
R_{pim}	0.011 (0.330)	0.012 (0.311)	0.014 (0.299)	0.018 (0.312)
Completeness (%)	100.0 (100.0)	100.0 (100.0)	100.0 (100.0)	100.0 (100.0)
$< I/\sigma(I) >$	27.2 (1.7)	24.9 (2.0)	22.3 (2.1)	18.1 (2.0)
$CC_{\frac{1}{2}}$	1.000 (0.745)	0.999 (0.778)	0.999 (0.793)	0.999 (0.756)

	$\frac{1}{4} - 720^\circ$	$\frac{1}{2} - 360^\circ$	$1 - 180^\circ$
Crystal parameters			
Space group	$I2_13$	$I2_13$	$I2_13$
Unit-cell parameters (Å)	$a = b = c = 78.12$	$a = b = c = 78.11$	$a = b = c = 78.11$
Data statistics			
Resolution range (Å)	39.06-1.40 (1.42-1.40)	39.06-1.40 (1.42-1.40)	39.05-1.40 (1.42-1.40)
No. of unique reflections	15788 (777)	15788 (777)	15788 (777)
Multiplicity	75.2 (64.2)	37.6 (31.9)	18.8 (16.0)
R_{merge}	0.078 (1.859)	0.064 (1.210)	0.053 (0.819)
R_{meas}	0.078 (1.874)	0.065 (1.229)	0.054 (0.846)
R_{pim}	0.009 (0.233)	0.010 (0.217)	0.012 (0.211)
Completeness (%)	100.0 (100.0)	100.0 (100.0)	100.0 (100.0)
$< I/\sigma(I) >$	34.3 (2.7)	29.9 (2.8)	26.8 (2.9)
$CC_{\frac{1}{2}}$	1.000 (0.858)	1.000 (0.873)	0.999 (0.887)

	$\frac{1}{2} - 720^\circ$	$1 - 360^\circ$
Crystal parameters		
Space group	$I2_13$	$I2_13$
Unit-cell parameters (Å)	$a = b = c = 78.12$	$a = b = c = 78.11$
Data statistics		
Resolution range (Å)	55.24-1.40 (1.42-1.40)	39.05-1.40 (1.42-1.40)
No. of unique reflections	15789 (777)	15788 (777)
Multiplicity	75.2 (64.2)	37.6 (31.9)
R_{merge}	0.065 (1.293)	0.055 (0.855)
R_{meas}	0.066 (1.303)	0.056 (0.869)
R_{pim}	0.007 (0.162)	0.009 (0.153)
Completeness (%)	100.0 (100.0)	100.0 (100.0)
$< I/\sigma(I) >$	42.4 (3.9)	37.0 (4.1)
$CC_{\frac{1}{2}}$	1.000 (0.923)	0.999 (0.938)

	$1 - 720^\circ$
Crystal parameters	
Space group	$I2_13$
Unit-cell parameters (Å)	$a = b = c = 78.11$
Data statistics	
Resolution range (Å)	55.24-1.40 (1.42-1.40)
No. of unique reflections	15789 (777)
Multiplicity	75.1 (64.1)
R_{merge}	0.057 (0.909)
R_{meas}	0.058 (0.916)
R_{pim}	0.007 (0.114)
Completeness (%)	100.0 (100.0)
$< I/\sigma(I) >$	50.5 (5.7)
$CC_{\frac{1}{2}}$	1.000 (0.965)

Table S3. *Merging statistics for all combinations of subsets of data and digital transmissions for insulin crystal 3*

	$\frac{1}{8} - 720°$	$\frac{1}{4} - 360°$	$\frac{1}{2} - 180°$	$1 - 90°$
Crystal parameters				
Space group	$I2_13$	$I2_13$	$I2_13$	$I2_13$
Unit-cell parameters (Å)	$a = b = c = 78.05$	$a = b = c = 78.05$	$a = b = c = 78.04$	$a = b = c = 78.05$
Data statistics				
Resolution range (Å)	55.19-1.40 (1.42-1.40)	55.19-1.40 (1.42-1.40)	55.18-1.40 (1.42-1.40)	39.02-1.40 (1.42-1.40)
No. of unique reflections	15741 (787)	15741 (787)	15724 (782)	15739 (787)
Multiplicity	75.4 (65.0)	37.7 (32.4)	18.8 (16.2)	9.4 (8.1)
R_{merge}	0.100 (2.362)	0.080 (1.567)	0.067 (1.065)	0.055 (0.698)
R_{meas}	0.101 (2.380)	0.081 (1.591)	0.069 (1.099)	0.058 (0.746)
R_{pim}	0.012 (0.294)	0.013 (0.278)	0.016 (0.273)	0.019 (0.261)
Completeness (%)	100.0 (100.0)	100.0 (100.0)	100.0 (100.0)	100.0 (100.0)
$< I/\sigma(I) >$	26.2 (2.0)	23.8 (2.2)	20.9 (2.4)	19.1 (2.4)
$CC_{\frac{1}{2}}$	1.000 (0.806)	0.999 (0.790)	0.999 (0.807)	0.999 (0.794)

	$\frac{1}{4} - 720°$	$\frac{1}{2} - 360°$	$1 - 180°$
Crystal parameters			
Space group	$I2_13$	$I2_13$	$I2_13$
Unit-cell parameters (Å)	$a = b = c = 78.05$	$a = b = c = 78.05$	$a = b = c = 78.04$
Data statistics			
Resolution range (Å)	55.19-1.40 (1.42-1.40)	55.19-1.40 (1.42-1.40)	39.02-1.40 (1.42-1.40)
No. of unique reflections	15741 (787)	15741 (787)	15740 (787)
Multiplicity	75.4 (65.0)	37.6 (32.4)	18.8 (16.1)
R_{merge}	0.084 (1.657)	0.068 (1.104)	0.059 (0.751)
R_{meas}	0.084 (1.670)	0.069 (1.122)	0.061 (0.776)
R_{pim}	0.010 (0.206)	0.011 (0.196)	0.014 (0.193)
Completeness (%)	100.0 (100.0)	100.0 (100.0)	100.0 (100.0)
$< I/\sigma(I) >$	32.5 (3.0)	29.4 (3.2)	25.5 (3.4)
$CC_{\frac{1}{2}}$	1.000 (0.884)	1.000 (0.896)	0.999 (0.887)

	$\frac{1}{2} - 720°$	$1 - 360°$
Crystal parameters		
Space group	$I2_13$	$I2_13$
Unit-cell parameters (Å)	$a = b = c = 78.05$	$a = b = c = 78.04$
Data statistics		
Resolution range (Å)	39.03-1.40 (1.42-1.40)	39.02-1.40 (1.42-1.40)
No. of unique reflections	15748 (767)	15740 (787)
Multiplicity	75.3 (64.4)	37.6 (32.4)
R_{merge}	0.072 (1.166)	0.061 (0.782)
R_{meas}	0.072 (1.175)	0.062 (0.794)
R_{pim}	0.008 (0.146)	0.010 (0.139)
Completeness (%)	100.0 (100.0)	100.0 (100.0)
$< I/\sigma(I) >$	40.4 (4.4)	35.6 (4.7)
$CC_{\frac{1}{2}}$	1.000 (0.935)	0.999 (0.939)

	$1 - 720°$
Crystal parameters	
Space group	$I2_13$
Unit-cell parameters (Å)	$a = b = c = 78.05$
Data statistics	
Resolution range (Å)	55.19-1.40 (1.42-1.40)
No. of unique reflections	15741 (787)
Multiplicity	75.3 (64.9)
R_{merge}	0.064 (0.823)
R_{meas}	0.064 (0.829)
R_{pim}	0.007 (0.103)
Completeness (%)	100.0 (100.0)
$< I/\sigma(I) >$	48.4 (6.5)
$CC_{\frac{1}{2}}$	1.000 (0.969)

Table S4. *Merging statistics for all combinations of subsets of data and digital transmissions for insulin crystal 4*

C.4. Resolution Limits for Digitally Attenuated Data

The more extremely attenuated data in Section 3.4 where the attenuation factor reached 4^{-6} (*i.e.* $\frac{1}{4096}$) are described in full in Table S5. As may be seen from the results, the overall $I/\sigma(I)$ drops rapidly, though not by a factor of ~ 2 as may be expected as the resolution limit is also falling, and the merging statistics become rapidly worse. On the other hand, the data *are* correctly processed even with a tiny fraction of the scattered photons, so the data could in principle give insight into the

crystal system with very little radiation damage.

Digital transmission	4^0	4^{-1}	4^{-2}	4^{-3}
Crystal parameters				
Space group	$I2_13$	$I2_13$	$I2_13$	$I2_13$
Unit-cell parameters (Å)				
$a = b = c$	78.12	78.11	78.12	78.12
Data statistics				
Resolution range (Å)	55.24-1.29 (1.31-1.29)	39.06-1.36 (1.38-1.36)	39.06-1.46 (1.49-1.46)	55.24-1.59 (1.62-1.59)
No. of unique reflections	20129 (999)	17197 (855)	13955 (704)	10830 (528)
Multiplicity	33.0 (10.4)	36.5 (21.6)	38.0 (37.6)	38.3 (38.3)
R_{merge}	0.054 (2.117)	0.077 (2.374)	0.115 (2.217)	0.188 (2.052)
R_{meas}	0.055 (2.228)	0.078 (2.431)	0.117 (2.248)	0.191 (2.079)
R_{pim}	0.009 (0.679)	0.013 (0.518)	0.019 (0.366)	0.031 (0.335)
Completeness (%)	100.0 (99.9)	100.0 (100.0)	100.0 (100.0)	100.0 (100.0)
$< I/\sigma(I) >$	31.3 (0.9)	22.9 (1.1)	16.9 (1.4)	11.6 (0.9)
$CC_{\frac{1}{2}}$	1.000 (0.378)	1.000 (0.589)	1.000 (0.698)	0.999 (0.621)
Digital transmission	4^{-4}	4^{-5}	4^{-6}	
Crystal parameters				
Space group	$I2_13$	$I2_13$	$I2_13$	
Unit-cell parameters (Å)				
$a = b = c$	78.12	78.13	78.14	
Data statistics				
Resolution range (Å)	55.24-1.78 (1.81-1.78)	39.07-1.94 (1.97-1.94)	55.25-2.15 (2.19-2.15)	
No. of unique reflections	7751 (375)	6021 (309)	4458 (215)	
Multiplicity	36.8 (32.5)	28.2 (20.3)	16.7 (9.6)	
R_{merge}	0.312 (1.457)	0.479 (1.307)	0.595 (1.135)	
R_{meas}	0.316 (1.480)	0.487 (1.340)	0.614 (1.195)	
R_{pim}	0.051 (0.258)	0.087 (0.291)	0.143 (0.365)	
Completeness (%)	100.0 (100.0)	100.0 (100.0)	100.0 (100.0)	
$< I/\sigma(I) >$	7.8 (0.9)	4.9 (0.8)	3.5 (0.9)	
$CC_{\frac{1}{2}}$	0.998 (0.638)	0.990 (0.517)	0.857 (0.368)	

Table S5. *Merging statistics to a resolution limit approximately determined by the* $CC_{\frac{1}{2}} \sim 0.5$.

Appendix D
Cubic Insulin / Multiple Crystals

D.1. Combining Partial Data

Partial data sets from the data from the four crystals of cubic insulin were combined with `xia2` / `DIALS` - all permutations of the first 360°, 180° and 90° of each data set were processed (*i.e.* six combinations of two crystals, four of three and one of all four) with the results in the following tables. Pairs of 360° may be compared with each of the full 720° sets from a single crystal.

Crystals (×360°)	1+2	1+3	1+4	2+3	2+4	3+4
Crystal parameters						
Space group	I$2_1$3	I$2_1$3	I$2_1$3	I$2_1$3	I$2_1$3	I$2_1$3
Unit-cell parameters (Å)	$a = b = c = 78.09$	$a = b = c = 78.11$	$a = b = c = 78.08$	$a = b = c = 78.09$	$a = b = c = 78.06$	$a = b = c = 78.08$
Data statistics						
Resolution range (Å)	55.22-1.40 (1.42-1.40)	39.06-1.40 (1.42-1.40)	55.21-1.40 (1.42-1.40)	39.04-1.40 (1.42-1.40)	55.19-1.40 (1.42-1.40)	55.21-1.40 (1.42-1.40)
No. of unique reflections	15781 (799)	15788 (777)	15781 (799)	15780 (799)	15749 (767)	15749 (767)
Multiplicity	75.1 (63.7)	75.1 (64.1)	75.1 (63.1)	75.1 (63.5)	75.2 (64.4)	75.2 (64.3)
R_{merge}	0.054 (0.811)	0.055 (0.850)	0.059 (0.812)	0.056 (0.822)	0.060 (0.789)	0.061 (0.826)
R_{meas}	0.055 (0.817)	0.055 (0.857)	0.060 (0.819)	0.056 (0.828)	0.060 (0.795)	0.061 (0.833)
R_{pim}	0.006 (0.102)	0.006 (0.107)	0.007 (0.103)	0.006 (0.103)	0.007 (0.099)	0.007 (0.103)
Completeness (%)	100.0 (100.0)	100.0 (100.0)	100.0 (100.0)	100.0 (100.0)	100.0 (100.0)	100.0 (100.0)
$< I/\sigma(I) >$	54.8 (6.2)	53.6 (5.9)	50.6 (6.3)	53.1 (6.2)	51.0 (6.7)	49.2 (6.4)
$CC_{\frac{1}{2}}$	1.000 (0.969)	1.000 (0.969)	1.000 (0.968)	1.000 (0.972)	1.000 (0.971)	1.000 (0.966)

Crystals (×360°)	1+2+3	1+2+4	1+3+4	2+3+4
Crystal parameters				
Space group	I$2_1$3	I$2_1$3	I$2_1$3	I$2_1$3
Unit-cell parameters (Å)	$a = b = c = 78.10$	$a = b = c = 78.08$	$a = b = c = 78.09$	$a = b = c = 78.07$
Data statistics				
Resolution range (Å)	39.05-1.40 (1.42-1.40)	55.21-1.40 (1.42-1.40)	55.22-1.40 (1.42-1.40)	39.04-1.40 (1.42-1.40)
No. of unique reflections	15780 (799)	15781 (799)	15781 (799)	15748 (767)
Multiplicity	112.6 (95.9)	112.6 (94.5)	112.6 (95.3)	112.8 (96.8)
R_{merge}	0.055 (0.828)	0.058 (0.806)	0.059 (0.829)	0.059 (0.816)
R_{meas}	0.055 (0.832)	0.059 (0.810)	0.059 (0.833)	0.059 (0.820)
R_{pim}	0.005 (0.085)	0.005 (0.083)	0.005 (0.085)	0.006 (0.083)
Completeness (%)	100.0 (100.0)	100.0 (100.0)	100.0 (100.0)	100.0 (100.0)
$< I/\sigma(I) >$	65.9 (7.5)	63.3 (7.8)	62.2 (7.6)	62.0 (7.9)
$CC_{\frac{1}{2}}$	0.999 (0.979)	1.000 (0.979)	1.000 (0.980)	1.000 (0.977)

Crystals (×360°)	1+2+3+4
Crystal parameters	
Space group	I$2_1$3
Unit-cell parameters (Å)	$a = b = c = 78.08$
Data statistics	
Resolution range (Å)	55.21-1.40 (1.42-1.40)
No. of unique reflections	15781 (799)
Multiplicity	150.1 (126.7)
R_{merge}	0.058 (0.820)
R_{meas}	0.058 (0.823)
R_{pim}	0.005 (0.073)
Completeness (%)	100.0 (100.0)
$< I/\sigma(I) >$	72.9 (8.9)
$CC_{\frac{1}{2}}$	1.000 (0.983)

Table S6. *Merging statistics for combinations of* 360° *data sets from crystals 1, 2, 3, 4 of cubic insulin.*

Crystals (×180°)	1+2	1+3	1+4	2+3	2+4	3+4
Crystal parameters						
Space group	I$2_1$3	I$2_1$3	I$2_1$3	I$2_1$3	I$2_1$3	I$2_1$3
Unit-cell parameters (Å)	$a = b = c = 78.09$	$a = b = c = 78.11$	$a = b = c = 78.08$	$a = b = c = 78.09$	$a = b = c = 78.05$	$a = b = c = 78.07$
Data statistics						
Resolution range (Å)	39.04-1.40 (1.42-1.40)	55.23-1.40 (1.42-1.40)	55.21-1.40 (1.42-1.40)	39.04-1.40 (1.42-1.40)	55.19-1.40 (1.42-1.40)	55.21-1.40 (1.42-1.40)
No. of unique reflections	15780 (799)	15789 (777)	15781 (799)	15780 (799)	15741 (787)	15749 (767)
Multiplicity	37.5 (31.7)	37.5 (32.1)	37.5 (31.4)	37.5 (31.7)	37.6 (32.3)	37.5 (32.0)
R_{merge}	0.053 (0.774)	0.053 (0.809)	0.058 (0.777)	0.053 (0.795)	0.058 (0.765)	0.059 (0.801)
R_{meas}	0.053 (0.787)	0.053 (0.822)	0.059 (0.790)	0.054 (0.807)	0.059 (0.777)	0.059 (0.813)
R_{pim}	0.009 (0.139)	0.009 (0.145)	0.009 (0.140)	0.009 (0.143)	0.009 (0.136)	0.010 (0.143)
Completeness (%)	100.0 (100.0)	100.0 (100.0)	100.0 (100.0)	100.0 (100.0)	100.0 (100.0)	100.0 (100.0)
$< I/\sigma(I) >$	39.9 (4.5)	39.2 (4.2)	36.5 (4.6)	38.8 (4.4)	36.9 (4.8)	35.5 (4.6)
$CC_{\frac{1}{2}}$	0.999 (0.945)	1.000 (0.945)	1.000 (0.942)	0.999 (0.949)	1.000 (0.945)	1.000 (0.938)

Crystals (×180°)	1+2+3	1+2+4	1+3+4	2+3+4
Crystal parameters				
Space group	I$2_1$3	I$2_1$3	I$2_1$3	I$2_1$3
Unit-cell parameters (Å)	$a = b = c = 78.10$	$a = b = c = 78.07$	$a = b = c = 78.09$	$a = b = c = 78.07$
Data statistics				
Resolution range (Å)	55.22-1.40 (1.42-1.40)	55.21-1.40 (1.42-1.40)	55.22-1.40 (1.42-1.40)	55.20-1.40 (1.42-1.40)
No. of unique reflections	15781 (799)	15749 (767)	15781 (799)	15749 (767)
Multiplicity	56.3 (47.8)	56.3 (48.2)	56.3 (47.5)	56.3 (48.2)
R_{merge}	0.053 (0.795)	0.057 (0.776)	0.057 (0.798)	0.057 (0.791)
R_{meas}	0.053 (0.803)	0.057 (0.785)	0.057 (0.806)	0.058 (0.799)
R_{pim}	0.007 (0.116)	0.008 (0.113)	0.008 (0.117)	0.008 (0.115)
Completeness (%)	100.0 (100.0)	100.0 (100.0)	100.0 (100.0)	100.0 (100.0)
$< I/\sigma(I) >$	48.3 (5.5)	45.7 (5.8)	45.0 (5.5)	45.0 (5.7)
$CC_{\frac{1}{2}}$	1.000 (0.964)	0.999 (0.962)	0.999 (0.961)	1.000 (0.962)

Crystals (×180°)	1+2+3+4
Crystal parameters	
Space group	I$2_1$3
Unit-cell parameters (Å)	$a = b = c = 78.08$
Data statistics	
Resolution range (Å)	39.04-1.40 (1.42-1.40)
No. of unique reflections	15780 (799)
Multiplicity	75.0 (63.1)
R_{merge}	0.056 (0.791)
R_{meas}	0.057 (0.797)
R_{pim}	0.006 (0.100)
Completeness (%)	100.0 (100.0)
$< I/\sigma(I) >$	52.9 (6.4)
$CC_{\frac{1}{2}}$	1.000 (0.973)

Table S7. *Merging statistics for combinations of* 180° *data sets from crystals 1, 2, 3, 4 of cubic insulin.*

Crystals (×90°)	1+2	1+3	1+4	2+3	2+4	3+4
Crystal parameters						
Space group	$I2_13$	$I2_13$	$I2_13$	$I2_13$	$I2_13$	$I2_13$
Unit-cell parameters (Å)	$a = b = c = 78.09$	$a = b = c = 78.11$	$a = b = c = 78.08$	$a = b = c = 78.08$	$a = b = c = 78.05$	$a = b = c = 78.07$
Data statistics						
Resolution range (Å)	39.04-1.40 (1.42-1.40)	55.23-1.40 (1.42-1.40)	39.04-1.40 (1.42-1.40)	55.21-1.40 (1.42-1.40)	39.03-1.40 (1.42-1.40)	55.20-1.40 (1.42-1.40)
No. of unique reflections	15780 (799)	15789 (777)	15780 (799)	15781 (799)	15740 (787)	15749 (767)
Multiplicity	18.7 (15.8)	18.7 (16.0)	18.7 (15.7)	18.7 (15.7)	18.7 (16.2)	18.7 (16.0)
R_{merge}	0.051 (0.794)	0.053 (0.832)	0.056 (0.759)	0.054 (0.825)	0.056 (0.757)	0.057 (0.794)
R_{meas}	0.053 (0.820)	0.054 (0.859)	0.057 (0.784)	0.055 (0.853)	0.057 (0.781)	0.059 (0.820)
R_{pim}	0.012 (0.205)	0.012 (0.214)	0.013 (0.196)	0.013 (0.213)	0.013 (0.194)	0.013 (0.204)
Completeness (%)	100.0 (100.0)	100.0 (100.0)	100.0 (100.0)	100.0 (100.0)	100.0 (100.0)	100.0 (100.0)
$< I/\sigma(I) >$	28.2 (3.1)	26.7 (2.9)	26.4 (3.2)	26.7 (2.9)	26.7 (3.4)	25.3 (3.1)
$CC_{\frac{1}{2}}$	0.999 (0.891)	1.000 (0.877)	0.999 (0.886)	0.999 (0.874)	0.999 (0.885)	1.000 (0.878)

Crystals (×90°)	1+2+3	1+2+4	1+3+4	2+3+4
Crystal parameters				
Space group	$I2_13$	$I2_13$	$I2_13$	$I2_13$
Unit-cell parameters (Å)	$a = b = c = 78.09$	$a = b = c = 78.07$	$a = b = c = 78.08$	$a = b = c = 78.07$
Data statistics				
Resolution range (Å)	55.22-1.40 (1.42-1.40)	55.20-1.40 (1.42-1.40)	55.21-1.40 (1.42-1.40)	55.20-1.40 (1.42-1.40)
No. of unique reflections	15781 (799)	15749 (767)	15781 (799)	15749 (767)
Multiplicity	28.1 (23.8)	28.1 (24.1)	28.1 (23.7)	28.1 (24.1)
R_{merge}	0.053 (0.824)	0.055 (0.778)	0.056 (0.801)	0.056 (0.801)
R_{meas}	0.054 (0.842)	0.056 (0.795)	0.057 (0.818)	0.057 (0.818)
R_{pim}	0.010 (0.172)	0.010 (0.161)	0.011 (0.167)	0.011 (0.166)
Completeness (%)	100.0 (100.0)	100.0 (100.0)	100.0 (100.0)	100.0 (100.0)
$< I/\sigma(I) >$	33.3 (3.6)	32.8 (4.0)	31.7 (3.9)	31.9 (3.9)
$CC_{\frac{1}{2}}$	1.000 (0.921)	0.999 (0.921)	1.000 (0.924)	1.000 (0.921)

Crystals (×90°)	1+2+3+4
Crystal parameters	
Space group	$I2_13$
Unit-cell parameters (Å)	$a = b = c = 78.08$
Data statistics	
Resolution range (Å)	55.21-1.40 (1.42-1.40)
No. of unique reflections	15781 (799)
Multiplicity	37.4 (31.4)
R_{merge}	0.056 (0.804)
R_{meas}	0.056 (0.817)
R_{pim}	0.009 (0.145)
Completeness (%)	100.0 (100.0)
$< I/\sigma(I) >$	37.3 (4.4)
$CC_{\frac{1}{2}}$	0.999 (0.943)

Table S8. *Merging statistics for combinations of* 90° *data sets from crystals 1, 2, 3, 4 of cubic insulin.*

Appendix E
CDK2 results

E.1. Merging Statistics

Sample 1 Transmission (%)	$\frac{1}{64}$	$\frac{1}{16}$	$\frac{1}{4}$	1	4	16	64
Crystal parameters							
Space group	P 21 21 21	P 21 21 21	P 21 21 21	P 21 21 21	P 21 21 21	P 21 21 21	P 21 21 21
Unit-cell parameters (Å)							
a	53.47	53.48	53.48	53.53	53.51	53.51	53.59
b	71.94	71.96	71.96	72.01	71.99	72.01	72.16
c	72.33	72.36	72.38	72.39	72.35	72.33	72.40
Data statistics							
Resolution range (Å)	42.91-1.84 (1.87-1.84)	42.93-1.64 (1.67-1.64)	42.92-1.50 (1.52-1.50)	42.96-1.41 (1.43-1.41)	72.35-1.33 (1.35-1.33)	72.33-1.29 (1.31-1.29)	72.40-1.30 (1.32-1.30)
No. of unique reflections	24928 (1180)	35012 (1681)	45528 (2191)	54697 (2648)	64923 (3131)	71026 (3431)	69770 (3394)
Multiplicity	12.7 (13.2)	12.7 (12.8)	12.5 (12.7)	12.4 (12.3)	12.3 (12.0)	12.2 (11.2)	12.2 (11.6)
R_{merge}	0.255 (1.770)	0.159 (1.886)	0.114 (1.852)	0.081 (1.817)	0.087 (2.225)	0.080 (1.319)	0.088 (1.069)
R_{meas}	0.266 (1.842)	0.166 (1.965)	0.119 (1.930)	0.084 (1.896)	0.091 (2.322)	0.084 (1.384)	0.091 (1.119)
R_{pim}	0.074 (0.503)	0.046 (0.544)	0.033 (0.538)	0.024 (0.535)	0.025 (0.658)	0.024 (0.410)	0.026 (0.325)
Completeness (%)	100.0 (96.8)	100.0 (97.6)	100.0 (97.6)	100.0 (98.8)	100.0 (98.4)	100.0 (98.1)	100.0 (98.1)
$< I/\sigma(I) >$	5.8 (1.0)	7.9 (1.1)	9.9 (1.2)	13.1 (1.1)	12.6 (0.9)	12.3 (0.9)	11.0 (0.9)
$CC_{\frac{1}{2}}$	0.994 (0.625)	0.998 (0.604)	0.999 (0.557)	0.999 (0.602)	0.999 (0.642)	0.998 (0.753)	0.998 (0.755)
Sample 2 Transmission (%)	$\frac{1}{64}$	$\frac{1}{16}$	$\frac{1}{4}$	1	4	16	64
Crystal parameters							
Space group	P 21 21 21	P 21 21 21	P 21 21 21	P 21 21 21	P 21 21 21	P 21 21 21	P 21 21 21
Unit-cell parameters (Å)							
a	53.43	53.43	53.43	53.44	53.45	53.47	53.54
b	71.94	71.95	71.95	71.96	71.97	72.01	72.10
c	72.49	72.51	72.52	72.53	72.55	72.58	72.65
Data statistics							
Resolution range (Å)	42.89-1.66 (1.69-1.66)	43.01-1.48 (1.51-1.48)	42.90-1.33 (1.35-1.33)	43.02-1.23 (1.25-1.23)	72.55-1.16 (1.18-1.16)	72.58-1.13 (1.15-1.13)	72.65-1.15 (1.17-1.15)
No. of unique reflections	33787 (1645)	47371 (2294)	65003 (3188)	81912 (4036)	96812 (4321)	103344 (4117)	99426 (4284)
Multiplicity	12.8 (12.9)	12.7 (12.2)	12.5 (12.3)	12.3 (11.5)	11.7 (6.7)	11.2 (5.2)	11.4 (6.0)
R_{merge}	0.188 (1.732)	0.111 (1.678)	0.074 (2.080)	0.051 (1.936)	0.038 (1.587)	0.034 (1.159)	0.044 (1.061)
R_{meas}	0.195 (1.802)	0.116 (1.751)	0.078 (2.170)	0.053 (2.026)	0.039 (1.720)	0.035 (1.288)	0.046 (1.164)
R_{pim}	0.054 (0.495)	0.032 (0.496)	0.022 (0.613)	0.015 (0.590)	0.011 (0.643)	0.010 (0.540)	0.013 (0.462)
Completeness (%)	100.0 (99.2)	100.0 (99.5)	100.0 (99.7)	100.0 (99.7)	99.4 (90.1)	98.0 (79.1)	99.0 (86.4)
$< I/\sigma(I) >$	7.5 (0.8)	11.2 (1.1)	14.3 (1.0)	18.5 (1.1)	22.3 (1.1)	26.0 (1.1)	20.2 (0.9)
$CC_{\frac{1}{2}}$	0.998 (0.554)	0.999 (0.589)	1.000 (0.528)	1.000 (0.573)	1.000 (0.548)	1.000 (0.595)	0.999 (0.599)
Sample 3 Transmission (%)	$\frac{1}{64}$	$\frac{1}{16}$	$\frac{1}{4}$	1	4	16	64
Crystal parameters							
Space group	P 21 21 21	P 21 21 21	P 21 21 21	P 21 21 21	P 21 21 21	P 21 21 21	P 21 21 21
Unit-cell parameters (Å)							
a	53.38	53.38	53.39	53.39	53.40	53.43	53.50
b	72.03	72.03	72.03	72.04	72.05	72.09	72.16
c	72.32	72.32	72.33	72.33	72.35	72.37	72.43
Data statistics							
Resolution range (Å)	42.89-1.67 (1.70-1.67)	42.89-1.46 (1.48-1.46)	42.89-1.33 (1.35-1.33)	42.89-1.22 (1.24-1.22)	72.05-1.16 (1.18-1.16)	72.37-1.13 (1.15-1.13)	72.43-1.16 (1.18-1.16)
No. of unique reflections	33097 (1619)	49245 (2433)	64820 (3157)	83691 (4147)	96973 (4563)	104080 (4457)	97358 (4608)
Multiplicity	12.8 (12.9)	12.7 (11.8)	12.6 (12.4)	12.3 (10.9)	11.7 (6.4)	11.1 (4.7)	11.5 (6.1)
R_{merge}	0.214 (1.614)	0.134 (1.996)	0.088 (2.013)	0.062 (1.948)	0.046 (1.426)	0.042 (1.103)	0.053 (1.090)
R_{meas}	0.223 (1.681)	0.140 (2.087)	0.092 (2.101)	0.064 (2.045)	0.048 (1.552)	0.044 (1.235)	0.055 (1.189)
R_{pim}	0.062 (0.465)	0.039 (0.605)	0.026 (0.596)	0.018 (0.616)	0.014 (0.598)	0.013 (0.540)	0.016 (0.463)
Completeness (%)	100.0 (100.0)	100.0 (99.8)	100.0 (99.3)	100.0 (99.6)	99.8 (95.0)	99.0 (85.9)	99.8 (95.6)
$< I/\sigma(I) >$	6.9 (0.9)	9.5 (0.9)	12.7 (1.1)	15.5 (1.1)	19.7 (1.1)	20.1 (1.0)	16.9 (1.0)
$CC_{\frac{1}{2}}$	0.997 (0.601)	0.999 (0.506)	0.999 (0.500)	1.000 (0.532)	1.000 (0.556)	1.000 (0.552)	0.998 (0.621)
Sample 4 Transmission (%)	$\frac{1}{64}$	$\frac{1}{16}$	$\frac{1}{4}$	1	4	16	64
Crystal parameters							
Space group	P 21 21 21	P 21 21 21	P 21 21 21	P 21 21 21	P 21 21 21	P 21 21 21	P 21 21 21
Unit-cell parameters (Å)							
a	53.36	53.36	53.36	53.36	53.37	53.41	53.50
b	72.00	72.00	72.00	72.01	72.02	72.06	72.18
c	72.38	72.39	72.39	72.39	72.40	72.44	72.55
Data statistics							
Resolution range (Å)	42.87-1.69 (1.72-1.69)	42.95-1.47 (1.49-1.47)	42.87-1.34 (1.36-1.34)	42.87-1.22 (1.24-1.22)	72.02-1.16 (1.18-1.16)	72.06-1.12 (1.14-1.12)	72.18-1.19 (1.21-1.19)
No. of unique reflections	31986 (1585)	48232 (2358)	63370 (3075)	83670 (4088)	96790 (4431)	105471 (3939)	90674 (4273)
Multiplicity	12.8 (13.0)	12.7 (11.8)	12.6 (12.3)	12.4 (11.1)	11.8 (6.7)	11.2 (4.9)	11.9 (8.8)
R_{merge}	0.215 (1.360)	0.132 (1.772)	0.087 (1.957)	0.063 (1.975)	0.047 (1.394)	0.043 (1.213)	0.060 (1.313)
R_{meas}	0.224 (1.415)	0.138 (1.853)	0.090 (2.042)	0.065 (2.070)	0.049 (1.511)	0.045 (1.356)	0.062 (1.396)
R_{pim}	0.062 (0.388)	0.038 (0.535)	0.025 (0.576)	0.018 (0.615)	0.014 (0.565)	0.013 (0.587)	0.018 (0.465)
Completeness (%)	100.0 (99.2)	100.0 (98.3)	100.0 (98.6)	100.0 (98.5)	99.6 (92.1)	97.6 (73.7)	99.9 (96.3)
$< I/\sigma(I) >$	6.5 (0.9)	9.2 (0.9)	12.1 (1.1)	14.4 (1.1)	18.7 (1.1)	18.4 (1.0)	16.3 (1.2)
$CC_{\frac{1}{2}}$	0.997 (0.671)	0.999 (0.523)	0.999 (0.524)	1.000 (0.533)	1.000 (0.491)	0.999 (0.487)	0.998 (0.703)

Table S9. *Merging statistics for crystals 1 - 4 of CDK2, to resolution limits determined from $CC_{\frac{1}{2}} \sim 0.5$ with transmissions from $\frac{1}{64}$ to 64%.*

E.2. Molecular Replacement

Molecular Replacement for CDK2 data sets was performed with `PHASER` (McCoy *et al.*, 2007) using as a search model PDB entry 1HCK(Schulze-Gahmen *et al.*, 1996) which has the same symmetry but the unit cell axes permuted. The following script was used, with the sequence derived from the input PDB file:

```
phaser << eof
title MR with CDK2 from 1HCK
mode MR_AUTO
hklin AUTOMATIC_DEFAULT_free.mtz
labin F=F SIGF=SIGF
ensemble cdk2 PDB CDK2_1hck.pdb IDENTITY 100
```

```
composition protein sequence CDK2.seq num 1
search ensemble cdk2 num 1
root CDK2
eof
```

In all cases a very strong solution was found, perhaps unremarkable considering that the model is approximately identical to the structure, however noteworthy as the data as presented in Table S9 have a very low $I/\sigma(I)$ in many cases.

References

Diederichs, K. (2006). *Acta Crystallographica Section D*, **62**(1), 96–101.

Evans, P. R. & Murshudov, G. N. (2013). *Acta Crystallogr. D. Biol. Crystallogr.* **69**(Pt 7), 1204–14.

McCoy, A. J., Grosse-Kunstleve, R. W., Adams, P. D., Winn, M. D., Storoni, L. C. & Read, R. J. (2007). *Journal of Applied Crystallography*, **40**(4), 658–674.

Schulze-Gahmen, U., De Bondt, H. L. & Kim, S.-H. (1996). *Journal of Medicinal Chemistry*, **39**(23), 4540–4546. PMID: 8917641.
